# Cardiogenic Shock Management in the Modern Era: A Narrative Review of Percutaneous Mechanical Circulatory Support Devices

**DOI:** 10.3390/jcdd13010009

**Published:** 2025-12-22

**Authors:** Srijit Jana, Makayla Wijesinghe, Michael V. DiCaro, KaChon Lei, Nazanin Houshmand, Chowdhury Ahsan

**Affiliations:** 1 Department of Internal Medicine, Las Vegas School of Medicine, University of Nevada, Las Vegas, NV 89102, USA; srijitjana8@gmail.com; 2 Reno School of Medicine, University of Nevada, Reno, NV 89102, USA; makaylawijesinghe@med.unr.edu; 3 Department of Cardiology, Las Vegas School of Medicine, University of Nevada, Las Vegas, NV 89102, USA; nazanin.houshmand@unlv.edu (N.H.); chahsan92@yahoo.com (C.A.)

**Keywords:** cardiogenic shock, shock, mechanical circulatory support

## Abstract

Cardiogenic shock (CS) remains a significant clinical challenge with persistently high mortality rates. Defined by impaired cardiac output resulting in end-organ hypoperfusion, CS commonly arises from acute myocardial infarction (AMI-CS) or acute exacerbations of heart failure (HF-CS). The severity of CS is classified by the Society for Cardiovascular Angiography and Interventions (SCAI) into stages A (at risk) through E (extremis), which informs treatment strategies, including pharmacotherapy and mechanical circulatory support (MCS). Recent advancements in percutaneous mechanical circulatory support devices, including intra-aortic balloon pumps (IABPs), Impella devices, TandemHeart, Protek-Duo, and veno-arterial extracorporeal membrane oxygenation (VA-ECMO), have transformed management paradigms by offering targeted hemodynamic support. While DanGer-SHOCK, a pivotal randomized trial, demonstrated improved outcomes with early Impella use in anterior STEMI-associated CS, the trial’s focus population and center expertise suggest that its findings should be interpreted in the context of broader AMI-CS and HF-CS presentations. Device selection is guided by shock severity, anatomical considerations, comorbidities, and institutional capabilities. This review synthesizes current evidence, evaluates the clinical utility and efficacy of existing and emerging percutaneous MCS technologies, and highlights ongoing clinical trials and future directions in optimizing CS management. Emphasis is placed on individualized patient selection, evidence-based deployment of MCS devices, and multidisciplinary team collaboration, which collectively represent a critical transition towards improving clinical outcomes in CS.

## 1. Introduction

Cardiogenic shock (CS) is defined as a state of end-organ hypoperfusion caused by left ventricular (LV), right ventricular (RV), or biventricular (BiV) dysfunction. Compromised performance of the myocardium, valves, conduction system, or pericardium results in reduced cardiac output (CO), leading to the biochemical, mechanical, and clinical findings observed in CS [1]. The diagnosis of CS is reached through a combination of clinical characteristics and objective findings. Objectively, CS is defined by the presence of systolic blood pressure (SBP) ≤ 90 mmHg for at least 30 min or requiring vasopressors to maintain SBP above 90 or mean arterial pressure (MAP) above 60 mmHg. Clinically, CS presents with signs and symptoms of poor tissue perfusion due to low CO, commonly manifesting as an uncomfortable patient with cool and mottled extremities, jugular venous distension, peripheral edema, dyspnea, reduced urine output (UOP), and tachycardia [2].

CS is most commonly caused by acute cardiac events, such as myocardial infarction (AMI-CS) or an acute exacerbation of congestive heart failure (HF-CS). Other etiologies include myocarditis, massive pulmonary embolism (PE), pericarditis, arrhythmias, or valvular disease. LV dysfunction manifests as decreased compliance (reduced end-diastolic volume) or decreased contractility (reduced stroke volume), both of which impair ventricular function and reduce CO. RV dysfunction can occur independently, or as a consequence of LV dysfunction, and can manifest as reduced ventricular compliance, increased ventricular dilation, or increased RV afterload. Decreased left ventricular end-diastolic pressure (LVEDP) may result from intraventricular septal bowing from increased pressures in the RV, compounding systemic hypoperfusion [3,4].

When evaluating for shock, objective data are crucial. Patients experiencing CS will have objective evidence of end-organ damage, including elevated lactate, abnormal liver function tests, and impaired kidney function. Patients will also have poor UOP despite classically appearing volume overloaded. In addition, pro-inflammatory markers, such as Cystatin C and Interleukin-6, as well as B-type Natriuretic Peptide (BNP), are incorporated into the CLIP score, which predicts 30-day mortality in patients with AMI-CS. Although the score provides valuable prognostic information, its clinical utility is limited because these biomarkers are not routinely measured in practice, and the score has not been validated in non-AMI causes of CS [5]. Signs of CS, including peripheral edema, jugular venous distension, S3 gallop, tricuspid/mitral regurgitation murmurs, pulmonary edema, hepatomegaly, hypotension, and hypoperfusion, may be present and can act as markers for evaluating CS. In severe stages of CS, altered mental status, oliguria, and mottled skin may be seen [6].

CS continues to carry a mortality rate of 40% to 60%, with hospital mortality remaining between 27% and 51%. Prior to the introduction of percutaneous coronary interventions (PCIs) in the 1950s, CS carried a mortality rate as high as 80% [1]. Although PCI has reduced mortality by nearly 50% in AMI-CS, outcomes remain poor [7]. AMI-CS occurs in 5–10% of all AMIs, with a higher risk in STEMI, elderly (>75 years), and female patients [7]. Isolated RV shock accounts for 3–5% of CS, while BiV involvement is seen in up to 50% of cases. HF-CS has a lower mortality (~20%) but still poses significant clinical challenges [7]. Acute decompensated heart failure (HF) is the underlying cause of about one in five cases of CS. Increased SCAI shock stage is directly correlated with higher mortality. Although hospitalizations of CS increased between 2004 and 2018, inpatient mortality rates have steadily declined [7].

The purpose of mechanical circulatory support (MCS) in CS is to preserve adequate tissue perfusion in the presence of low baseline CO, with the end goal being a bridge to recovery or a bridge to destination therapy. MCS attempts to achieve this by targeting different hemodynamic parameters. Veno-Arterial Extracorporeal Membrane Oxygenation (VA-ECMO) and TandemHeart can reduce LVEDP, while other devices, such as micro-axial flow pumps, aim to reduce LV afterload and enhance CO [1,2]. Historically, MCS has been utilized to manage severe SCAI stages of CS. However, as CS mortality remains unacceptably high, the field stands at the threshold of a new era wherein early, targeted MCS deployment may improve outcomes. Early recognition and timely initiation of MCS significantly improve the outcomes of patients. Survival rates when MCS is implemented within 1.25 h of shock onset are notably higher: 66% compared to 26% for the delayed intervention [8]. The pivotal DanGer-SHOCK trial further reinforced this paradigm shift, demonstrating that early Impella CP support improved 180-day survival in carefully selected patients primarily presenting with anterior STEMI-related CS [9]. Numerous other trials are also ongoing, signifying a rapid shift in clinical management. This review aims to synthesize the latest evidence, critically examine device-specific roles, review updated guidelines on management, and provide a contemporary perspective on the integration of MCS in clinical practice. The literature discussed was identified through targeted searches of PubMed, supplemented by manual review of reference lists from key studies.

Targeted management of CS can be achieved by first differentiating the etiology (LV, RV, or BiV dysfunction). Pulmonary artery catheterization (PAC) can assist in the evaluation by providing hemodynamic data using a Swan–Ganz Catheter, which can determine pulmonary capillary wedge pressure (PCWP), right arterial pressure (RAP), cardiac index (CI), cardiac power output (CPO), and pulmonary artery pulsatility index (PAPi). These hemodynamic parameters guide MCS deployment and selection in order to optimize treatment and outcomes in CS. Important hemodynamic data are outlined in Appendix A [10].

## 2. Cardiogenic Shock: Definitions and Phenotypes

### 2.1. Overview of Classic Definitions

While CS has a variety of etiologies, the two phenotypes that will primarily be discussed are CS secondary to AMI-CS and HF-CS. AMI-CS occurs as a result of disrupted blood flow to the myocardium, usually due to atherosclerotic plaque rupture resulting in a type 1 myocardial infarction [1]. The myocardium becomes ischemic and eventually loses its functional capability, causing a loss of CO and resultant hemodynamic consequences, which define CS: a CI of less than 2.2, SBP ≤ 90 mmHg for at least 30 min, PCWP > 15, and signs of end-organ damage. Revascularization remains the first-line treatment for AMI-CS [11]. The CULPRIT-SHOCK trial demonstrated a 17% absolute reduction in the primary endpoint of 30-day mortality or renal replacement therapy with PCI of the culprit vessel lesion in patients with AMI-CS [12].

HF-CS occurs due to a reduction in myocardial contractility, either de novo or from an exacerbation of pre-existing heart failure. Contractile dysfunction results in reduced CO, hypotension, systemic hypoperfusion, and tissue hypoperfusion [13]. The loss of CO results in compensatory mechanisms and neurohormonal cascades, including tachycardia, activation of the Renin–Angiotensin–Aldosterone pathway (RAAS), and catecholamine surge, which progressively worsen the stage of shock [3]. HF-CS can be further classified by the underlying cause of myocardial dysfunction, which may include acute myocarditis, takotsubo or peripartum cardiomyopathy, alcohol or tachycardia-induced cardiomyopathy, excessive beta-blockade, septic cardiomyopathy, or infiltrative diseases like amyloidosis. LV ejection fraction (LVEF) may be reduced or preserved depending on the etiology [14]. HF-CS may not initially present with hypotension; instead, it will usually show signs of hypoperfusion early in the presentation. Signs of hypoperfusion include cold extremities, alteration in baseline mental status, decreasing UOP, and rising lactic acid levels from baseline. Additionally, HF-CS manifests with fluid overload, including pulmonary and peripheral edema. Compared to patients in AMI-CS, patients in HF-CS tend to be younger, have a lower LVEF, and lower CPO [10,15].

Potential hemodynamic presentations of CS can be categorized as warm, cold, dry, or wet based on physical examination and invasive hemodynamic parameters. Cold and wet CS is classic CS and presents with reduced CI, increased systemic vascular resistance index (SVRI), and increased PCWP. Clinically, patients present with signs of volume overload and hypoperfusion. Cold and dry CS, known as euvolemic CS, presents with reduced CI, increased SVRI, and normal PCWP. Vasodilatory or mixed CS presents with reduced CI, low or normal SVRI, and increased PCWP. Warm and dry shock is typically not classified as cardiogenic in nature; it typically presents with increased CI, low or normal SVRI, and low PCWP. Warm and dry shock is generally associated with septic or distributive shock [1].

CS can exhibit changes in the pressure–volume relationship during the cardiac cycle. Normally, the pressure–volume loop of the cardiac cycle consists of isovolumetric contraction, ejection, isovolumetric relaxation, and filling [16]. Both acute and chronic heart failure can shift the entire pressure–volume loop to the right and decrease the width of the loop, indicating increased filling pressures and decreased stroke volume. The end-diastolic pressure–volume relationship (EDPVR) depicts the relationship between ventricular filling pressures and full ventricular relaxation [4]. Chronic heart failure exhibits a further shift to the right than acute heart failure due to the longer duration of remodeling. This is reflected by the rightward shift of the EDPVR. Generally, rightward shifts of the EDPVR are associated with systolic dysfunctions, whereas leftward shifts are associated with diastolic dysfunctions (hypertrophic cardiomyopathy, sarcoidosis, and other restrictive cardiomyopathies) [16,17]. These pressure–volume derangements translate clinically into elevated filling pressures, reduced effective forward flow, and progressive end-organ hypoperfusion, which define the hemodynamic substrate of CS and directly inform the need for early ventricular unloading and MCS escalation [18].

### 2.2. Society for Cardiovascular Angiography and Interventions (SCAI) Consensus Classification

SCAI classification has been used as the general classification system of CS based on hemodynamic parameters from PAC measurements and relevant laboratory values [19,20]. Appropriate staging and escalation are imperative in managing CS, given the relatively high mortality rate. With the creation of this classification, clinicians are able to make informed decisions on the escalation and de-escalation of pharmacotherapy and MCS deployment, respectively. The SCAI classification categorizes CS from Stages A to E, with Stage A being categorized as the least severe form of CS and E being the most severe and life-threatening form of CS.

Stage A (at risk): SCAI Stage A is defined as having risk factors for CS, such as myocardial infarction or acute exacerbation of chronic heart failure. Patients in SCAI Stage A do not have signs of hypotension and hypoperfusion and tend to have normal laboratory values. In this stage, medical management of the underlying conditions, HF or AMI, needs to be initiated. This includes utilizing guideline-directed medical therapy (GDMT) and diuresis for HF, or revascularization through PCI, and subsequently placing patients on a high-intensity statin and a dual antiplatelet regimen for AMI [19,20].

Stage B (Beginning): Patients in SCAI Stage B show early signs of hemodynamic instability, such as hypotension and tachycardia. However, laboratory values do not show signs of hypoperfusion (normal levels of lactate, aminotransferases, liver function tests, or renal function tests). In SCAI Stage B, treatment remains focused on treating the underlying etiology of CS. Clinicians may also consider an inotrope (e.g., dobutamine) or vasopressor (e.g., norepinephrine) trial. Furthermore, an MCS device, such as an IABP, can be considered for additional hemodynamic support [19].

Stage C (Classic): In SCAI Stage C, in addition to hemodynamic instability, signs of hypoperfusion are present and confirmed by laboratory findings, including elevated lactate ≥ 2 mmol/L, significantly abnormal renal function tests, aminotransferase levels, and liver function tests. This stage generally requires vasopressor or inotropic support to maintain MAPs of 60–65 mmHg. At this stage, an MCS device is also deployed to maintain hemodynamic stability. Device selection is based on the location of ventricular dysfunction (RV vs. LV vs. BiV) and the phenotype of CS (AMI vs. HF) [19].

Stage D (Deteriorating): In SCAI Stage D, shock is not improving despite escalating therapy in Stage C. Laboratory findings indicate worsening signs of hypoperfusion, such as elevated lactate levels ≥ 4 mmol/L, and worsening renal and liver function tests compared to previous stages. At this stage, patients often require higher doses of inotropes or vasopressors, along with escalation to mechanical circulatory support devices capable of delivering greater flow, potentially including VA-ECMO for full cardiopulmonary support [19].

Stage E (Extremis): In SCAI Stage E, patients are in imminent circulatory collapse despite aggressive pharmacotherapy and MCS support. Laboratory values signifying deleterious hypoperfusion include elevated lactate levels ≥ 8 mmol/L with signs of significant acidosis. Severe abnormalities in renal function, aminotransferase levels, and liver function tests are present as well. Patients at this stage are typically already on three different vasopressors, inotropes, or MCS devices. Full cardiopulmonary support with VA-ECMO is recommended at this stage, regardless of the CS phenotype or location of ventricular insult [19].

### 2.3. Clinical Utility and Prognostic Implications of SCAI Classification

The SCAI Consensus Classification of CS can be utilized to help diagnose the stage of CS and tailor therapy accordingly. Without timely and appropriate escalation of therapy, patients can deteriorate rapidly [1]. Despite advances in care, current 30-day mortality rates of AMI-CS are still high at 40–50%. Hemodynamic monitoring and labs, including PAC measurements, lactate levels, liver function tests, blood urea nitrogen (BUN), and creatinine (Cr), should be obtained at least every 6 h. Lactate levels remaining above 3.1 mmol/L after 8 h, or failure to show lactate clearance on serial measurements, are closely linked to higher mortality [21]. Additionally, PAC hemodynamic monitoring can help tailor treatment, particularly MCS device selection, by localizing ventricular abnormalities. A CPO measured less than 0.6 is seen in SCAI Stages C-E [22]. PAC profiles can also help differentiate shock subtypes:

- Left ventricular shock: PCWP > 15 mmHg, RA pressure < 15 mmHg, PAPi > 1.0 [23,24].
- Right ventricular shock: PCWP < 15 mmHg, RA pressure > 15 mmHg, PAPi < 1.0 [23,24].
- Biventricular shock: PCWP > 15 mmHg, RA pressure > 15 mmHg, PAPi < 1.0 [23,24].

Efforts should be made to wean vasopressors and/or MCS devices as laboratory parameters normalize and hemodynamic metrics improve based on PAC data and clinical status.

### 2.4. Risk Prediction Tools in CS

While MCS devices are now central to the management of CS, optimal timing for initiation and escalation, guided by shock severity, relevant laboratory parameters, and clinical presentation, remains an area for improvement. Accurate risk stratification is critical, as premature MCS deployment in patients who are unlikely to benefit from intervention may expose them to avoidable complications, such as hemolysis, acute kidney injury (AKI), or limb ischemia. Conversely, delayed initiation may result in hemodynamic support being offered when meaningful recovery is likely no longer achievable [18,23]. Risk stratification scores, such as the CLIP score, Cardiogenic Shock Score (CSS), IABP-SHOCK II score, the Japanese registry for Percutaneous Ventricular Assist Devices (J-PVAD) Score, AGEF Score, CardShock Score, and ECLS-SHOCK score, provide insight into the prognosis of patients suffering from CS and the indication of MCS deployment [24]. As described previously, the CLIP score utilizes pro-inflammatory markers, such as Cystatin C and Interleukin-6, and BNP, to predict 30-day mortality in patients with AMI-CS [5]. However, its applicability to non-AMI-CS is limited, and the required pro-inflammatory markers are not routinely measured in clinical practice [23].

Similarly, the IABP-SHOCK II score predicts 30-day mortality in AMI-CS using six variables: age > 73 years, prior stroke, admission glucose > 191 mg/dL, admission creatinine > 1.5 mg/dL, post-PCI TIMI flow < 3, and arterial lactate > 5 mmol/L. Each is assigned 1–2 points, with total scores classifying patients into low (0–2), intermediate (3–4), or high (5–9) risk [25]. Like the CLIP score, its applicability to non-AMI-CS is limited, while its advantages include utilizing easily obtained laboratory values in the clinical setting [18,23].

The J-PVAD and CSS scores aim to provide risk stratification in CS irrespective of etiology. The J-PVAD trial analyzed 2551 patients who required LV micro-axial flow pump support (Impella 2.5, 5.0, or CP) to maintain hemodynamic stability. The J-PVAD score incorporates 12 variables: age, sex, body mass index (BMI), fulminant myocarditis etiology, in-hospital cardiac arrest, VA-ECMO use, MAP, Cr, total bilirubin, lactate, lactate dehydrogenase (LDH), and albumin. Higher scores were associated with worse outcomes, with age ≥ 80 years, BMI ≥ 25.0 kg/m^2^, LDH ≥ 1000 IU/L, and Cr ≥ 2.0 mg/dL contributing most significantly to in-hospital mortality risk. The J-PVAD score offers the advantage of relying on readily obtainable laboratory values while assessing in-hospital mortality risk in CS regardless of etiology. However, its use is limited to cases managed with LV micro-axial flow pump support [18,24].

The CSS score, developed from a cohort of 934 patients, uses readily available point-of-care variables (age, sex, systolic blood pressure, heart rate, pH, lactate, glucose, and duration of CPR or cardiac arrest) to predict 30-day in-hospital mortality in CS regardless of etiology. Higher scores correlate with increased mortality risk. Similar to the J-PVAD score, it leverages easily obtainable clinical markers to guide MCS decision-making by balancing potential benefits against procedural risks and complications, but unlike the J-PVAD score, it is applicable beyond cases managed with micro-axial flow pumps. However, as it was derived from two tertiary care centers and relied solely on data available at initial presentation without comprehensive biomarker or hemodynamic inputs, its generalizability and predictive accuracy may be limited [18,23].

Similarly, the AGEF score utilizes patient age, estimated glomerular filtration rate (eGFR), and LVEF to determine 90-day mortality in patients with CS regardless of etiology. The scoring system is calculated by taking the patient’s age divided by the LVEF (%). An additional point is assigned if the creatinine clearance is below 60 mL/min. In the study design, the cutoff score of 2.26 was utilized to grade the mortality of the shock. AGEF scores greater than 2.26 were associated with a greater 90-day mortality, whereas scores lower than 2.26 were associated with lower 90-day mortality. A key advantage of the AGEF score is its simplicity, relying on readily available clinical variables and maintaining applicability across CS etiologies and independent of MCS strategy. The AGEF score was derived from pre-existing CardShock registry data, which lacked key clinical variables such as vasopressor requirements, infectious complications, and volume status—factors that may have introduced unmeasured confounding. Additionally, although the score demonstrated utility in predicting 90-day mortality, further studies are needed to validate its performance in short-term mortality prediction [18,26].

The CardShock Score estimates in-hospital 30-day mortality in patients with CS. It uses seven variables to estimate the risk of inpatient mortality: age > 75 years, altered mental status, previous MI or coronary artery bypass graft (CABG), active ACS, and LVEF < 40%. The maximum score possible is 9 points. An increased score is associated with worsened 30-day inpatient mortality. The advantage of this score, similar to the J-PVAD score and CSS, is its ability to predict mortality in both AMI-CS and non-AMI-CS. Additionally, it relies on easily obtainable clinical parameters to determine whether a patient may require escalation of therapy or initiation of MCS. However, despite its usefulness for predicting short-term outcomes, its utility for long-term mortality remains unclear, and the limited number of non-AMI-CS patients in the derivation studies reduces confidence in its accuracy for non-ischemic shock phenotypes [18,27].

The ECLS-SHOCK score was derived from the ECLS-SHOCK trial, which was developed to determine the 30-day inpatient mortality in severe AMI-CS. It utilizes six parameters to predict inpatient mortality, namely, age ≥ 69, female gender, ≥3 cardiovascular risk factors (i.e., Type II Diabetes Mellitus, hyperlipidemia, hypertension, smoking history, or known coronary artery disease (CAD)), baseline arterial lactate ≥ 7.1 mmol/L, and resuscitation efforts, for a maximum of seven points. Scores of 0–1 are classified as low risk, scores of 2–3 are classified as moderate risk, and scores of 4–7 are classified as severe risk. The ECLS-SHOCK score benefits from its use of readily available clinical variables to stratify risk in CS. However, its applicability is limited to AMI-CS, and its performance in non-AMI etiologies remains uncertain. Moreover, its stratification primarily reflects acute severity and may not reliably predict long-term mortality [18,28].

The CLIP, IABP-SHOCK II, J-PVAD, CSS, AGEF, CardShock, and ECLS-SHOCK scores (Table 1) offer structured frameworks for assessing the risks and benefits of MCS in CS, integrating pertinent laboratory findings and clinical variables. A consistent finding across these models is that markers of end-organ injury (elevated transaminases, lactate, and impaired renal function) are associated with worse outcomes in the setting of CS. Given the high mortality of CS, timely initiation and appropriate escalation of support remain critical. These scoring systems can assist clinicians in weighing the potential hemodynamic benefits of MCS against the procedural risks and device-related complications, thereby guiding more informed, individualized treatment decisions [18,23].

**Table 1:** CS risk prediction scores.

Risk Scores	Inputs	Predictive Use	Limitations
CLIP	Pro-inflammatory markers such as Cystatin C, Interleukin-6, and B-type Natriuretic Peptide (BNP) [5].	30-day mortality in AMI-CS [5].	Generalizability to non-AMI-CS is limited. Pro-inflammatory markers are not routinely measured in clinical practice [23].
IABP-SHOCK II	Age > 73 years, prior stroke, admission glucose >191 mg/dL, admission creatinine > 1.5 mg/dL, post-PCI TIMI flow < 3, and arterial lactate > 5 mmol/L [25].	30-day mortality in AMI-CS [25].	Generalizability to non-AMI-CS is limited [23].
J-PVAD	Age, sex, body mass index (BMI), fulminant myocarditis etiology, in-hospital cardiac arrest, VA-ECMO use, mean arterial pressure, creatinine, total bilirubin, lactate, LDH, and albumin [18,24].	In-hospital mortality regardless of etiology of CS. Specified in LV Impella support (2.5, 5.0, or CP) only [18,24].	Not generalizable to CS requiring non-LV Impella MCS devices [18,24].
CSS	Age, sex, systolic blood pressure, heart rate, pH, lactate, glucose, and duration of CPR or cardiac arrest [23].	30-day in-hospital mortality in CS regardless of etiology [23].	Generalizability and predictive accuracy may be limited [23].
AGEF	Age, estimated glomerular filtration rate (eGFR), and LVEF% [18,26].	90-day mortality in CS regardless of etiology [18,26].	Limited generalizability to short-term mortality (30-day mortality) [18,26].
CardShock	Age > 75 years, altered mental status, previous MI or coronary artery bypass graft (CABG), active ACS, and LVEF < 40% [18,27].	30-day inpatient mortality regardless of etiology [18,27].	Limited generalizability to long-term mortality in CS. Additionally, given the limited number of non-AMI-CS patients studied, may have less predictive value [18,27].
ECLS-SHOCK	Age ≥ 69, female gender, ≥3 cardiovascular risk factors, baseline arterial lactate ≥ 7.1 mmol/L, and resuscitation efforts [18,28].	30-day inpatient mortality in AMI-CS [18,28].	Not generalizable to non-AMI-CS. Cannot be utilized to accurately predict long-term mortality [18,28].

## 3. Percutaneous Mechanical Circulatory Support Devices: Technical Overview, Clinical Data, and Efficacy

MCS devices have become an increasingly integral component of CS management. A wide range of devices is currently in use, and novel technologies are under active investigation, aiming to improve safety profiles and expand clinical utility. This section provides an overview of commonly used percutaneous MCS devices, including the intra-aortic balloon pump (IABP), micro-axial flow pump, left ventricular assist device (LVAD), right ventricular assist device (RVAD), and Veno-Arterial Extracorporeal Membrane Oxygenation (VA-ECMO). In particular, we focus on their technical features, hemodynamic effects, current clinical data, indications, contraindications, and associated complications, as summarized in Table 2. Additionally, emerging devices, such as the PulseCath iVAC2L, NuPulse iVAS, Supira pVAD, and the Magenta pVAD, are reviewed in the context of ongoing research and clinical trials.

**Table 2:** Overview of MCS Devices.

Device Name	Indications	Flow Rates	Absolute Contraindications	Relative Contraindications	Device Complications
Intra-Aortic Balloon Pump (IABP)	HRPCI and Stage A-B AMI-CS and HF-CS [29]. Stage C LV AMI-CS and HF-CS [20,30].	0.5 L/min [29]	Aortic regurgitation, severe sepsis, aortic dissection, and aortic aneurysm [31].	Bleeding disorders, severe aortic calcifications, PAD, obstruction of left ventricular outflow tract (i.e., aortic stenosis), or arrhythmias including atrial fibrillation and, premature ventricular complexes (PVCs) [31].	Limb ischemia, vascular laceration, major hemorrhage, and iatrogenic aortic dissection [31]. Hemolysis, thrombocytopenia, cholesterol embolization, and, rarely, sepsis if the device remains in place for greater than 7 days [32,33,34].
Left Ventricular Impella Devices (CP, ECP, 2.5, 5.0, and 5.5)	HRPCI (CP, ECP, and 2.5) and Stage C-D LV AMI-CS and HF-CS (5.0, 5.5, and LD) [35,36,37].	2.5–6 L/min [35,36,37]	LV thrombus, mechanical aortic valve, severe aortic stenosis, LV outflow tract obstruction, severe aortic regurgitation, severe PAD, severe RV failure, atrial septal defects, ventral septal defects, LV rupture, and cardiac tamponade [37].	Access vessel diameter less than 6 mm, severe aortic calcification, and active insertion site infection [37].	Hemolysis, AKI, limb ischemia, and stroke [37,38,39,40].
Right Ventricular Impella Devices (RP and RP Flex)	Stage C-D RV CS (both AMI-CS and HF-CS) [20,29,30].	2–4 L/min [20,29,30]	Mechanical tricuspid valve or pulmonary valve prosthesis, severe tricuspid regurgitation, severe pulmonary regurgitation, and right heart thrombus or mass [37].	Active insertion site infection, severe PAD, pre-existing right-to-left shunt, and severe coagulopathy [41].	Hemolysis, AKI, limb ischemia, and stroke [37,38,39,40].
TandemHeart	HRPCI, Stage C-D LV AMI-CS, and HF-CS [42]. Can be utilized in the setting of severe mitral stenosis and possibly in the setting of aortic stenosis or LV thrombus as well [43,44,45].	3.5 to 5.0 L/min [42]	Right or left atrial thrombus, severe coagulopathies, severe aortic regurgitation, and severe ventral septal defect (VSD) [41].	Pre-existing renal impairment and active insertion site infection [46].	Bleeding, hemolysis, thrombosis, stroke, limb ischemia, access site infection, sepsis, device migration, atrial perforation, cardiac tamponade, air embolism during insertion, and iatrogenic residual ASD, resulting in right- to- left shunt [47].
VA-ECMO	Stage D-E refractory AMI-CS and HF-CS [19,30].	4–7 L/min [48,49,50,51]	Pre-existing terminal illness, aortic dissection or insufficiency, severe irreversible non-cardiopulmonary organ failure, or severe coagulopathy [52].	Advanced age (≥70 years), class III obesity, and active infection or sepsis [53].	Bleeding, limb ischemia, compartment syndrome, stroke, AKI, infection, and lung dysfunction [54,55].
Protek-Duo	Stage C-D RV-predominant CS (both AMI-CS and HF-CS) [56].	4–5 L/min [56]	Mechanical tricuspid valve or pulmonary valve prosthesis, severe tricuspid regurgitation, severe pulmonary regurgitation, and right heart thrombus or mass [57,58].	Severe coagulopathy and small body habitus [46,58].	Vascular injury, hemolysis, iatrogenic tricuspid regurgitation, pulmonary valve dysfunction, superior vena cava syndrome, cardiac wall perforation, pericardial effusion with tamponade, infection, embolism, thrombosis, and cannula migration [59].

### 3.1. Intra-Aortic Balloon Pump

**Figure 1:**
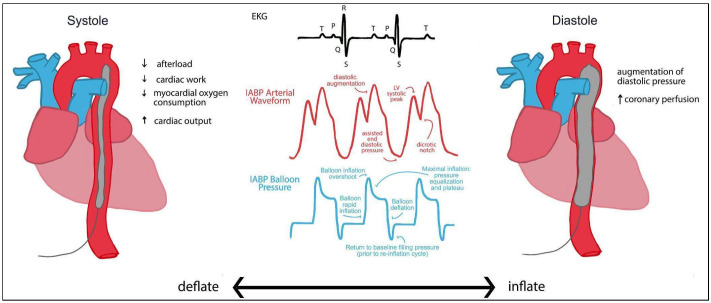
Intra-aortic balloon pump (IABP). The balloon deflates during systole and inflates during diastole. Inflation and deflation are timed relative to electrocardiogram and pressure waveforms. The P wave on the ECG represents atrial depolarization, the QRS complex represents ventricular repolarization, and the T wave represents atrial repolarization. The red waveform represents the intra-aortic balloon pump (IABP)–augmented arterial pressure waveform synchronized 1:1 with the ECG tracing. The blue waveform denotes balloon pressure, reflecting the inflation during diastole and deflation during systole. Open-access image adapted from Gillespie et al. The Intra-Aortic Balloon Pump: A Focused Review of Physiology, Transport Logistics, Mechanics, and Complications [65].

An IABP is a pulsatile form of MCS that has historically been used in AMI-CS in conjunction with revascularization via PCI. The device is a balloon-tipped catheter that is inserted into the proximal descending aorta via the femoral or axillary artery using a percutaneous approach. The balloon deflates during systole and inflates during diastole. By deflating during systole, a negative pressure system is created, pulling blood from the LV into the systemic circulation [60]. The negative pressure reduces afterload and improves CO, thus reducing myocardial oxygen requirements. As shown in Figure 1, Deflation is synchronized to the R wave of the electrocardiogram (ECG) or the upward stroke of the arterial pressure waveform. During diastole, the balloon inflates, pushing blood both anterograde and retrograde. Retrograde flow increases coronary perfusion by pushing blood against a closed aortic valve into the sinus of Valsalva. Balloon inflation coincides with the middle of the T wave of the ECG, as well as the dicrotic notch on the arterial pressure waveform [61].

IABPs remain the simplest, cost-effective, and easily deployable form of MCS due to their ease of bedside insertion and relatively favorable safety profile [29]. Weaning is achieved by reducing the ratio of the deflation–inflation cycle from 1:1 to 1:2 or 1:3, based on clinical improvement. On the pressure–volume (PV) loop, the entire loop shifts to the left on the EDPVR. Generally, the shape of the curve remains the same, indicating lower end-diastolic volume with preserved shape. Stroke volume increases as peak systolic pressures decrease due to systolic unloading during balloon deflation. However, the PV loop does not account for coronary perfusion during diastolic balloon inflation. Overall, an IABP decreases myocardial oxygen demand and provides hemodynamic support in CS [61].

Although randomized clinical trials such as IABP-SHOCK II failed to demonstrate a significant reduction in 30-day mortality, IABPs have been shown to improve hemodynamic measurements such as MAP, CO, and CPO [31]. It may also be used to improve cerebral perfusion in HF-CS patients with an LVEF less than 30% [62]. IABPs may be useful as a bridge to CABG in select patients. In high-risk PCI (HRPCI), typically defined as PCI in patients with multiple comorbidities, such as heart failure, kidney disease, or low LVEF, often involving complex coronary anatomy (i.e., chronic total occlusion, multivessel CAD) or urgent situations like CS, MCS devices are often required. Due to high-risk profiles and procedural complexity, pre-procedural planning is imperative in minimizing complications. In this setting, IABPs have been an effective standby method in providing hemodynamic support. The data from the Balloon Pump-Assisted Coronary Intervention Study (BCIS-1 trial) further support the use of IABPs as an adjunct support modality in HRPCI [61].

IABPs can provide meaningful hemodynamic support in early stages of CS (SCAI Stages A-B), with some studies suggesting a mortality benefit across shock phenotypes (AMI-CS and HF-CS) and ventricular pathologies [63]. Furthermore, the INOVA Shock Team considers that IABPs are a feasible option in SCAI Stage C LV-predominant shock, irrespective of etiology, although the overall evidence in this setting remains mixed [20,31].

Complications of IABPs occur in 6–25% of cases and are primarily vascular in nature. These include limb ischemia (necessitating regular checks of distal pulses), vascular laceration, major hemorrhage, and iatrogenic aortic dissection [64]. Non-vascular complications may include hemolysis, thrombocytopenia, cholesterol embolization, and, rarely, sepsis if the device remains in place for greater than 7 days [32,33,34]. Moreover, proper positioning of the balloon tip in the aorta, 2 cm distal to the left subclavian artery, is crucial. Misplacement can lead to limb or spinal cord ischemia, requiring device removal and reinsertion [29].

Absolute contraindications of IABPs include aortic regurgitation (compromising counterpulsation of the balloon pump), severe sepsis, aortic dissection, and aortic aneurysm. Relative contraindications include bleeding disorders, severe aortic calcifications, peripheral artery disease (PAD), obstruction of the left ventricular outflow tract (i.e., aortic stenosis), or arrhythmias, including atrial fibrillation and premature ventricular complexes (PVCs) [31]. Routine anticoagulation is not required for IABP support, though practice varies. UFH may be used, but studies show no differences in limb ischemia or bleeding with or without anticoagulation. Some centers reserve anticoagulation for the 1:2 or 1:3 modes [37].

### 3.2. Impella Devices (CP, 2.5, 5.0, 5.5, RP, and ECP)

**Figure 2:**
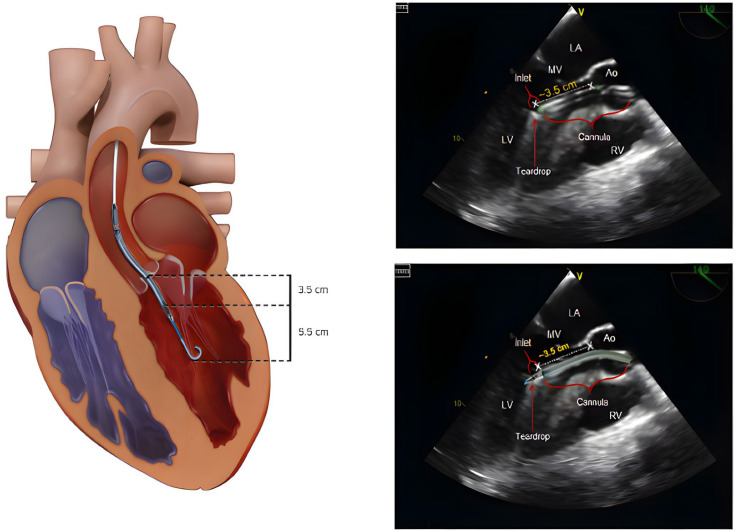
Left ventricle Impella positioning through the Aortic Outflow tract (red chamber). Left Atrium (LA), Mitral Valve (MV), Left Ventricle (LV), Aortic Outflow Tract (Ao), and Right Ventricle (RV) are visualized in the echocardiogram images. The 3.5 cm and 5.5 cm measurements are echocardiographic depth landmarks confirming correct Impella positioning, with the inlet below the aortic valve in the LV and the outlet in the ascending aorta. Open-access image obtained from Zein et al. A Review of the Impella Devices [36].

Impella devices are surgically or percutaneously implanted micro-axial rotary blood pumps that provide hemodynamic support for patients with CS or undergoing HRPCI. These pumps consist of an impeller, an integrated motor, and an inflow/outflow cannula, all mounted on a 9 French (Fr) catheter. By directly unloading ventricles, Impella devices are able to support CO and maintain MAP by promoting continuous forward flow of blood. Broadly, Impella devices can be categorized into LV and RV support systems. As demonstrated in Figure 2, LV micro-axial flow pumps, such as Impella 2.5, 5.0, 5.0, CP, ECP, and LD, draw blood from the LV into the ascending aorta, providing continuous blood flow independent of the cardiac cycle. Similarly, RV micro-axial flow pumps (Impella RP and RP flex) continuously deliver blood from the venous system, typically from the Inferior Vena Cava (IVC) or the internal jugular vein (IJV), into the pulmonary artery (PA), bypassing the RV during failure [35,36].

The Automated Impella Controller (AIC) manages device operation and troubleshooting, while SmartAssist technology allows for real-time hemodynamic monitoring, including measuring MAP, CPO, and LVEDP [35]. Impella devices demand an advanced level of technical skill due to the various insertion techniques and models. Impella 2.5 and CP models are inserted percutaneously, while Impella 5.5 requires surgical cutdown and a Dacron graft into the axillary or femoral artery for placement. This robust insertion process may require the involvement of cardiothoracic surgeons, interventional cardiologists, and teams that are proficient in the device’s hardware, including its catheter, purge system, and AIC. Additionally, proceduralists need to be trained in anticoagulation protocols and emergency complication management due to the higher incidence of complication rates [66].

Current anticoagulation guidelines for Impella micro-axial flow pumps vary. Recommended anticoagulation utilizes a heparin purge solution, typically consisting of 50 U/mL of 5% dextrose followed by systemic UFH with a general Activated Partial Thromboplastin Time (aPTT) goal of 40–60 s and an Activated Clotting Time (ACT) goal of 160–200 s, measured every 4–6 h. In the setting of heparin-induced thrombocytopenia (HIT), bivalirudin or argatroban can be utilized instead. However, anticoagulation protocols vary by institution, requiring further randomized clinical trials and studies to determine the optimal anticoagulation in patients who require Impella support [37].

Impella 2.5, which is inserted percutaneously through the common femoral artery or axillary artery, provides up to 2.5 L/min of forward blood flow and is typically used in HRPCI for up to 6 h and in CS up to 4 days. Impella CP is inserted percutaneously through the femoral artery and delivers up to 4.1 L/min (mean 3.3 L/min) flow through a 14 Fr pump and utilizes a fluid-filled sensor that measures aortic pressure as blood exits the pump [36,66]. Similar to Impella 2.5, Impella CP is also indicated for HRCPI and CS for up to 6 h and 4 days, respectively [36]. Impella ECP is a novel percutaneous transvalvular device that requires only a 9 Fr insertion sheath but can expand to 21 Fr, offering up to 5.5 L/min for short-term HRPCI and CS for up to 6 h and 4 days, respectively [37]. Impella 5.0 can be placed surgically or percutaneously and delivers up to 5.0 L/min blood flow during CS. It includes a pig-tailed catheter to ensure stability and can be used up to 14 days to manage CS. Impella 5.5 with SmartAssist is surgically placed in the aorta or axillary artery with a 21 Fr sheath. It provides an average 5.5 L/min flow rate, with a peak flow rate of 6 L/min, and utilizes a fiber-optic to modulate the flow rate [35,36].

RV support is provided by Impella RP, which is inserted percutaneously via the femoral vein and supports up to 4.0 L/min of blood flow from the IVC to the PA for up to 14 days [36]. Impella RP Flex adds versatility by being placed percutaneously in both the internal jugular vein and the femoral vein. When inserted via the IJV, it offloads blood from the superior vena cava (SVC) and RA to the PA; when inserted via the femoral vein, it offloads blood from the IVC [36]. Both RP devices support right heart circulation in cases of severe RV failure by directly bypassing the RV and unloading blood from the venous system directly into the pulmonary arterial system.

Impella devices primarily offer their hemodynamic benefit through direct ventricular unloading. In the LV, the unloading reduces LVEDP, PCWP, and afterload, while improving coronary and systemic perfusion. Although intrinsic myocardial stroke volume decreases due to reduced required workload, CO is maintained by the pump. The continuous unloading of the LV results in a leftward and downward shift of the PV loop due to loss of isovolumetric contraction and relaxation. As a result, this mechanism transforms the loop from its regular trapezoidal shape to a triangular shape, reflecting decreased myocardial oxygen requirements and improved energy efficiency. An Impella device does not augment the intrinsic myocardial contractility but instead reduces myocardial stress and promotes ventricular recovery [38,67]. Additionally, Impella devices can be combined for BiV support for “BiPella”, utilizing both LV and RV Impella pumps during BiV CS [68,69]. “EcPella”, the combination of LV Impella support with VA-ECMO, mitigates the increase in afterload typically induced by VA-ECMO alone, when full cardiopulmonary support is necessary. This integrated approach reduces myocardial oxygen demand and mitigates the risk of pulmonary congestion by constituting a form of LV venting [70,71].

Several studies and clinical trials have analyzed the effectiveness and efficacy of Impella deployment in managing CS. The most prominent of these studies includes the DanGer Shock Trial, which was an international, multicenter, randomized clinical trial that recruited 360 patients with anterior STEMI AMI-CS from Germany, Denmark, and the United Kingdom from January 2013 to June 2023. The trial studied the efficacy of Impella CP in the setting of anterior STEMI AMI-CS, revealing that Impella CP significantly reduced 180-day mortality compared to standard care alone. However, higher rates of adverse events, including bleeding, hemolysis, and limb ischemia, were noted in the Impella CP group compared to the standard care group [9]. The RECOVER Right Trial, a prospective, open-label, single-arm, non-randomized, multicenter study, examined the efficacy of Impella RP in 30 patients with CS secondary to RV failure. It found significant improvements in CI and central venous pressure with a 30-day survival rate of 73.3%. The ongoing STEMI-DTU trial is evaluating whether LV unloading with Impella CP, 30 min prior to PCI, reduces infarct size in patients with anterior STEMI [72,73]. PROTECT IV, another ongoing randomized trial, is assessing long-term outcomes of Impella-supported HRPCI in patients with reduced LVEF over the course of 3 years [72].

A large retrospective cohort analysis of 28,340 patients with AMI-CS by Dhruva et al. [74] demonstrated that, compared with IABPs, micro-axial Impella support was associated with higher in-hospital mortality and major bleeding, despite greater hemodynamic support. While micro-axial pumps have become widely incorporated into contemporary CS management, these findings highlight the ongoing controversy surrounding routine Impella use and reinforce the need for additional randomized trials to more clearly define their clinical benefit [74].

Registry data provide valuable insight into the utility of Impella devices in CS and HRPCI. However, because the data are derived from observational registries, the results cannot imply causality. The Cardiogenic Shock Working Group analyzed 6205 patients from 2020 to 2023, of whom 757 (12.5%) received Impella 5.0 or 5.5 in the setting of CS. Impella 5.0 or 5.5 was used as the sole device in 32%, while an Impella device was used in combination with other MCS devices for 68% of the patients receiving Impella support. Combined MCS devices had increased risks of limb ischemia, bleeding, and higher mortality. However, the overall survival-to-discharge rate was 67%, irrespective of whether Impella was used alone or in combination with other devices, with 45.5% successfully bridged to heart replacement therapy [75]. The limitations of the Cardiogenic Shock Working Group registry include its retrospective design, which introduces confounding bias, and the lack of standardized algorithms for MCS escalation. In addition, because patients frequently received combined MCS support, adverse events could not be temporally linked to any single device and were analyzed only in the context of the overall hospitalization [75]. The cVAD registry showed that pre-PCI Impella 2.5 placement for AMI-CS had superior 30-day survival in 154 patients compared to those who were treated with IABPs and/or inotropes [76]. Impella devices have become increasingly recognized as important components of contemporary CS management while still requiring cautious interpretation in the context of their complication profile.

Indications for Impella placement include HF-CS (particularly for RP, RP Flex, 5.0, 5.5, and LD), as well as HRPCI (particularly for 2.5, CP, and ECP). In SCAI Stage C-D LV HF-CS, Impella 5.5 can be utilized for durable hemodynamic support due to its ability to provide higher blood flow capacity and suitability for prolonged use [35,36,37]. In SCAI Stage C-D LV AMI-CS, Impella CP may be appropriate due to rapid percutaneous insertion without the need for surgical cutdown [35,36,37]. Whereas in SCAI Stage C RV HF-CS and RV AMI-CS, Impella RP can be utilized for right-sided support. In BiV HF-CS, Impella RP can be employed alongside Impella 5.5 for BiV support. In BiV AMI-CS, Impella CP can be considered for left-sided support instead of Impella RP [20,30].

Relative contraindications to LV Impella placement include an access vessel diameter less than 6 mm, severe aortic calcification, and active insertion site infection. Absolute contraindications include LV thrombus, the presence of a mechanical aortic valve, severe aortic stenosis, LV outflow tract obstruction (i.e., hypertrophic cardiomyopathy), severe aortic regurgitation, severe PAD, severe RV failure, atrial septal defects, ventral septal defects, LV rupture, and cardiac tamponade [37]. Contraindications to RV Impella placement include mechanical tricuspid valve or pulmonary valve prosthesis, severe tricuspid regurgitation, severe pulmonary regurgitation, right heart thrombus or mass, active insertion site infection, and severe coagulopathy [37,77]. General complications of Impella placement include hemolysis, AKI, limb ischemia, and stroke [37,38,39,40]. Hemolysis, typically caused by misplacement and improper suctioning, is identified by plasma-free hemoglobin levels (PfHgb) > 43 mg/dL on more than two measurements, taken 8 hours apart within 24 h of device placement, LDH elevation, and reduced haptoglobin [39].

### 3.3. TandemHeart

**Figure 3:**
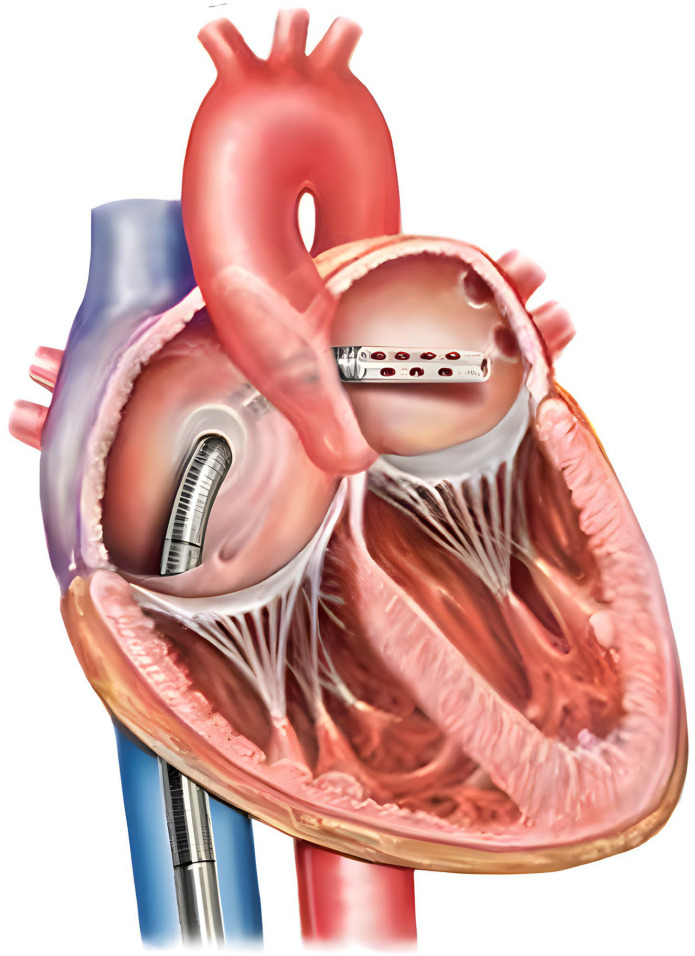
The TandemHeart system provides percutaneous MCS via transseptal cannulation, draining blood from the left atrium and returning it through an extracorporeal centrifugal pump to the systemic arterial circulation, thereby achieving left ventricular unloading and augmentation of forward flow. The large blue vessel represents the inferior vena cava, and the large red vessel represents the aorta. Open-access image obtained from Vranckx et al. The TandemHeart^®^, percutaneous transseptal left ventricular assist device: a safeguard in high-risk percutaneous coronary interventions. The six-year Rotterdam experience [85].

TandemHeart is a left-sided hemodynamic device that provides 3.5 to 5.0 L/min of blood through a centrifugal cardiac bypass device. It is commonly utilized in CS, decompensated HF, aortic and mitral valvulopathies, and HRPCI [42]. As exemplified in Figure 3, the device functions by drawing oxygenated blood from the left atrium (LA) through a transseptal cannula and inserting the blood into the common femoral artery at the level of the aortic bifurcation. The inflow cannula is a 21 Fr cannula that is inserted into the femoral vein and placed in the LA transseptally by fluoroscopic guidance [42]. Blood from the LA is then pumped into the outflow cannula, which is a 15–17 Fr catheter placed into the contralateral common femoral artery. If required, 12 Fr sheaths can be used in patients with a smaller femoral artery diameter. This configuration directly unloads the LA and improves hemodynamic parameters by decreasing LVEDP, PCWP, and right-sided filling pressures. However, given the slight increase in afterload due to retrograde blood flow, coronary perfusion is neutrally affected [78,79]. On the pressure–volume (PV) loop, stroke volume progressively narrows as flow rate increases, as blood is being shunted directly away from the LA and indirectly from the LV [80].

TandemHeart is the MCS device of choice in patients with severe mitral stenosis and can possibly be utilized in patients with aortic stenosis or LV thrombus as well [43,45,81]. It is generally utilized in SCAI Stage C-D LV-predominant CS, irrespective of the underlying CS phenotype (AMI vs. HF) [19,43,44,45]. It has also been proven to be quite a versatile system, due to its ability to be combined with Veno-Arterial ECMO (VA-ECMO) for Left Atrium Veno-Arterial ECMO (LAVA-ECMO). However, the device requires large-bore access for catheter insertion, resulting in increased bleeding risks [81]. Furthermore, the transseptal technique, required to introduce the catheter from the RA to the LA, is not widely performed. The transseptal puncture to place the 21 Fr inflow cannula in the LA with outflow via the femoral artery requires skilled operators and management of a centrifugal pump, hemodynamics, and anticoagulation [60]. Possible complications include bleeding, hemolysis, thrombosis, stroke, limb ischemia, access site infection, sepsis, device migration, atrial perforation, cardiac tamponade, air embolism during insertion, and iatrogenic residual ASD, resulting in right-to-left shunting [47]. Anticoagulation is necessary when utilizing TandemHeart to prevent thrombus formation or device thrombosis. Current guidelines suggest utilizing saline-based UFH, as dextrose-based UFH solutions may cause damage to the device motor. The ACT goal for insertion ranges from 300 to 400 s, while an ACT goal of 180–200 s is utilized for ongoing support. However, further data are required to study the long-term outcomes of anticoagulation in patients requiring TandemHeart support [37].

Earlier studies have shown the hemodynamic benefits of TandemHeart but have noted no improvement in short-term mortality rates. A randomized study of 41 patients completed in 2005 by Thiel et al. [82] compared management of AMI-CS between IABPs and TandemHeart. The TandemHeart arm had more favorable hemodynamic measurements with increased CPO, UOP, CO, and CI and decreased PCWP. However, patients who were placed on TandemHeart had more complications, with increased risk of limb ischemia and disseminated intravascular coagulation (DIC), and required blood transfusions, with no difference in 30-day mortality. Similarly, a 2006 randomized trial by Burkhoff et al. compared 42 patients in CS who received IABPs or TandemHeart. TandemHeart again improved hemodynamic parameters, such as increased CI and MAP and decreased PCWP, when compared to the IABP group. While the 30-day survival of the TandemHeart group was not statistically different, it was numerically higher than the IABP group (64% vs. 53%, respectively) [79,82].

In 2011, Kar et al. [83] conducted a prospective study of TandemHeart with 117 patients who were in CS refractory to IABPs and vasopressors. TandemHeart placement improved hemodynamic and metabolic indices, such as UOP, MAP, and CI, and decreased lactate levels. However, the 30-day mortality rate (43.3%) was reduced when compared to the mortality rate of the SHOCK trial (47%) [83]. The multicenter, prospective THEME registry evaluated 50 patients with CS who underwent TandemHeart implantation between May 2015 and June 2019. The study also demonstrated significant hemodynamic improvement following device support, including increases in CI and reductions in lactate levels [84]. Although limited by its observational design and lack of a comparator group, the registry reported 30-day and 180-day survival rates of 74% and 66%, respectively, suggesting that TandemHeart may provide meaningful support in selected patients with refractory CS. The limitations of the THEME registry include its small sample size and observational design, which preclude establishing causality [84]. Larger randomized controlled trials are needed to better define the magnitude and clinical relevance of the hemodynamic benefits observed [84].

Overall, TandemHeart has shown robust hemodynamic support and may be preferable to other MCS devices in specific clinical conditions. However, its complication profile, complexity of initiation, and lack of mortality benefit limit its widespread adoption.

### 3.4. Veno-Arterial Extracorporeal Membrane Oxygenation (VA-ECMO)

Veno-Arterial Extracorporeal Membrane Oxygenation (VA-ECMO) is a life-saving form of MCS for patients who require cardiopulmonary bypass. It can be used as a bridge to recovery or used for definitive therapies, such as left ventricular assist devices (LVADs) or heart transplantation (HT) [52,86]. VA-ECMO is generally considered to be the highest level of percutaneous MCS support [87]. Initiation of ECMO in severe stages of CS (Stage D or E) or post-cardiac arrest (cardiac arrest that is refractory to cardiopulmonary resuscitation) has been shown to prevent further circulatory collapse and improve chances of survival [30]. The increasing demand for ECMO is paving the way for new innovations and machinery.

**Figure 4:**
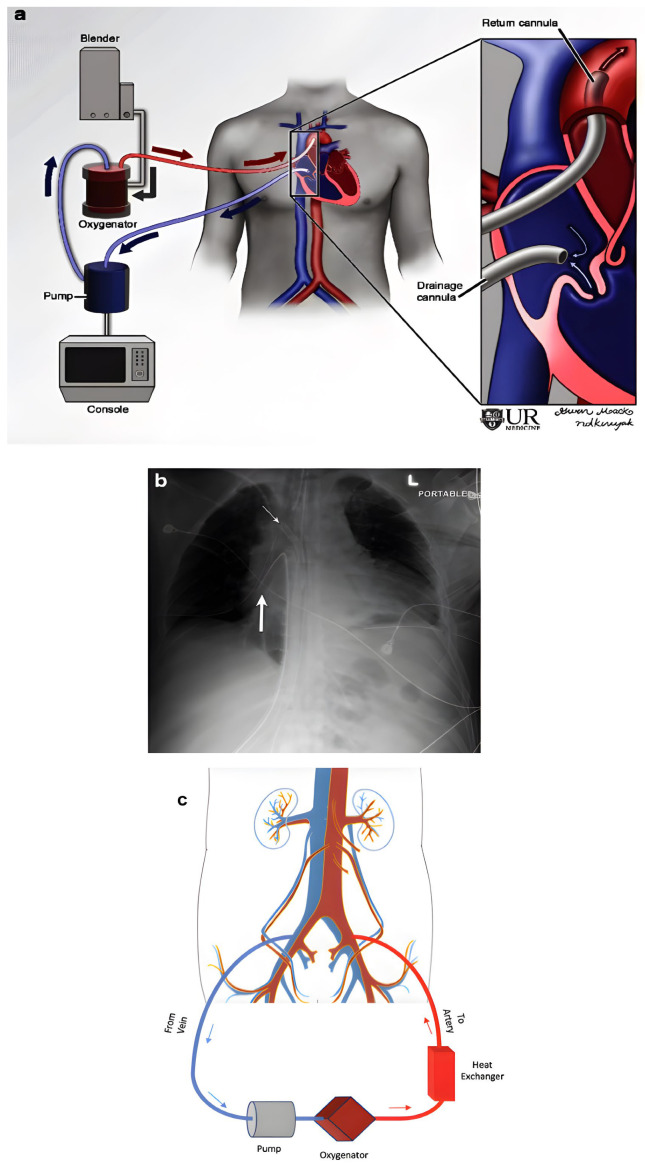
Veno-arterial extracorporeal membrane oxygenation (VA-ECMO) configuration. Panels (**a**) and (**b**) demonstrate a schematic illustration and corresponding chest radiograph of mediastinal VA-ECMO, respectively. Venous drainage is achieved via a cannula positioned in the right atrium (thick white arrow), with arterial return through a cannula in the ascending aorta (thin white arrow). Blue arrows indicate venous flow toward the oxygenator, while the red arrow denotes arterial return [97]. Illustration (**c**) exhibits the most common peripheral VA-ECMO cannulation strategy, with venous drainage via the femoral vein toward the right atrium (blue arrow) and arterial return via the femoral artery with antegrade flow to the descending aorta (red arrow) [98]. Open-access image obtained from Lee et al. Imaging Adults on Extracorporeal Membrane Oxygenation (ECMO).

VA-ECMO circuits are composed of three components: a centrifugal pump, oxygenator/heat exchange, and cannulae, as described in Figure 4. Contemporary oxygenators utilize hollow fiber polymethylpentene, allowing for efficient heat and gas exchange. This integrated process enables simultaneous oxygenation and heat exchange, reducing the device’s surface area and priming volume while minimizing inflammation and hemolysis. Cannulae, composed of polyurethane to minimize blood interaction, are inserted percutaneously or surgically. While Venous–Venous ECMO (VV-ECMO) is typically reserved for severe respiratory failure only, VA-ECMO is used to treat both severe CS and respiratory failure [88]. VA-ECMO drains blood from the venous system (typically the IVC) through the oxygenator and heat exchanger and then retrogradely returns it to the arterial system (e.g., distal abdominal aorta or common femoral artery).

VA-ECMO effectively unloads the LV, reducing LVEDP and preload, but it increases afterload due to the retrograde flow of blood into the arterial system [89]. This increased afterload can precipitate pulmonary edema, lung injury, and LV thrombus formation secondary to elevated left-sided filling pressures. To further verify thrombus formation, echocardiograms can visualize a “smoke sign”, indicating LV blood stasis [89,90]. In order to decrease afterload, LV micro-axial flow pumps or IABPs can be deployed to further enhance unloading, constituting LV venting [89,91]. VA-ECMO can be initiated peripherally at the bedside or centrally via surgical cannulation. The latter requires a specialized multidisciplinary team. Clinicians must be capable of managing afterload increases through timely LV unloading, weaning from ECMO, and transitioning patients to long-term LVAD support or HT, if irreversible cardiac damage is present [60].

Blood flow through the ECMO circuit generally follows Poiseuille’s Law. Percutaneous VA-ECMO typically uses a 19–25 Fr venous cannula and a 15–19 Fr arterial cannula [86]. VA-ECMO can be maintained for several days to weeks as a bridge to LVAD placement or HT. Cannulation strategies include femoral vein and femoral artery (peripheral), right atrium and central aorta (central), or internal jugular vein and axillary artery configurations. The peripheral configuration (femoral vein to femoral artery) is the most rapidly deployed form of VA-ECMO under ultrasound guidance, commonly performed at bedside. However, the peripheral configuration carries increased rates of limb ischemia. North–South Syndrome, or Harlequin syndrome, also occurs more frequently in the peripheral configuration as the brain and upper body receive hypoxic blood due to the retrograde blood flow [92]. Conversely, the lower body and extremities tend to receive more oxygenated blood. The central cannulation configuration, requiring sternotomy, can be utilized as a way to mitigate North–South Syndrome. Additionally, the central cannulation can be escalated from the peripheral configuration when insufficient hemodynamic support is provided, and it is less likely to cause limb ischemia compared to peripheral cannulation. A modified configuration, VAV-ECMO, introduces an additional outflow cannula into the IJV, improving oxygenation and mitigating North–South Syndrome [93].

Anticoagulation is essential during VA-ECMO to prevent oxygenator microthrombi and systemic thromboembolism. Unfractionated heparin (UFH) is the most commonly used agent, with bivalirudin or argatroban reserved for patients with concern for HIT. UFH is typically initiated with a 50–100 U/kg bolus followed by a maintenance infusion of approximately 10 U/kg/h. Per the 2017 ELSO guidelines, the recommended ACT target is 180–220 s. Additional monitoring strategies include aPTT (1.5–2.5 × baseline) and anti-Xa levels (0.3–0.7 IU/mL) to ensure adequate anticoagulation. Further studies are needed to define optimal anticoagulation targets and monitoring strategies for patients supported with VA-ECMO [37].

Hemodynamically, VA-ECMO progressively shifts the PV loop to the right and upwards along the EDPVR curve with increasing flow rates. This shift is due to the increases in LVEDP and preload caused by the retrograde aortic blood flow and disrupted LV ejection. Stroke volume progressively decreases with increasing VA-ECMO flow rates, as the MCS device supports the CO [94]. VA-ECMO is indicated in AMI, myocarditis, cardiotoxic drugs, end-stage dilated cardiomyopathy, postpartum cardiomyopathy, post-cardiotomy or post-transplantation shock, and advanced stages of CS. Recent uses include massive PE causing significant RV failure, stress-induced cardiomyopathy, and sepsis-induced cardiomyopathy [88]. Contraindications to VA-ECMO placement include pre-existing terminal illness, aortic dissection or insufficiency, severe irreversible non-cardiopulmonary organ failure, or severe coagulopathy [52]. Complications include bleeding, limb ischemia, compartment syndrome, stroke, AKI, infection, and lung dysfunction [54,55].

VA-ECMO is indicated in cardiac arrest refractory to CPR, with the Extracorporeal Life Support Organization (ESLO) recommending VA-ECMO after 10 to 15 min of unsuccessful resuscitation. While VA-ECMO is commonly utilized in SCAI Stage D or E CS regardless of underlying etiology or ventricular pathology, institutional guidelines may vary [95,96]. Currently, per the European Society of Cardiology, VA-ECMO carries a Class IIa recommendation, with Level C evidence [48]. Despite the device’s physiological benefits, the present data on mortality benefits remain mixed.

The ECMO-CS Trial, a multicenter randomized clinical trial performed in the Czech Republic, enrolled 122 patients from September 2014 to January 2022 to investigate the difference between early and delayed VA-ECMO deployment in CS management [48]. After excluding 5 patients due to lack of consent, 58 patients were placed in an immediate VA-ECMO group and 59 in a standard-of-care group, with VA-ECMO implementation only upon deterioration. The primary endpoint of the study was the composite of death from any cause, resuscitated cardiac arrest, and additional MCS implementation at 30 days, showing no statistical difference between the two groups. Notably, 39% of the standard care patients eventually required VA-ECMO [48]. A 2023 meta-analysis by Zeymer et al. similarly found no significant difference in 30-day mortality in 567 patients (284 VA-ECMO, 283 controls) when comparing patients treated with VA-ECMO and those who were treated with standard care protocols in AMI-CS. An increased risk of device complications, such as bleeding and limb ischemia, was more common in the VA-ECMO group [49]. These findings underscore the need for careful patient selection and consideration of device-related complications when using VA-ECMO in CS, given its mixed mortality benefits.

ECLS-SHOCK was a randomized, multicenter trial that recruited 420 patients, of whom only 417 participated, to determine if VA-ECMO showed a 30-day survival benefit in AMI-CS patients for whom early revascularization was planned. Conducted in Germany and Slovenia, patients were recruited from June 2019 to November 2022. A total of 209 patients received VA-ECMO and 208 received medical management, where the median age was 63 years, and 81% of the patients were male. Approximately two-thirds presented with STEMI, with 47.6% showing LAD occlusion. PCI was used for revascularization in 96.6% of the cases, and most patients were classified as SCAI Stage C–E shock. The study did not find any significant reduction in the 30-day mortality rate between the VA-ECMO group and the control group. However, there was an increased risk of complications in the VA-ECMO group, including bleeding, limb ischemia, and stroke or thromboembolic events [50]. ECLS-SHOCK purports that VA-ECMO usage in advanced stages of CS (SCAI Stages C-D) may not improve outcomes compared to standard medical therapy alone, given the device’s risk profile. Thus, clinicians must perform a nuanced risk–benefit assessment of initiating VA-ECMO in patients with AMI-CS, tailoring the decision based on the clinical presentation of each individual patient. Additionally, risk stratification scores may help guide clinical decision-making regarding treatment escalation in AMI-CS [50].

Similarly, EURO SHOCK was a randomized clinical trial that studied the efficacy of using VA-ECMO after revascularization in AMI-CS patients. However, an IABP was implemented if patients in the medical therapy group experienced severe hemodynamic instability. An IABP was also included in the VA-ECMO group as a form of LV venting. From January 2020 to January 2022, 35 patients with AMI-CS were recruited; 18 patients received standard therapy, and 17 patients received VA-ECMO with an IABP. The 30-day all-cause mortality was numerically reduced but not statistically significantly reduced in the VA-ECMO group compared to the standard therapy group. Additionally, all-cause mortality at 12 months was also numerically lower in the VA-ECMO group. However, similar to the findings of ECLS-SHOCK, there was a significantly increased risk of bleeding and limb ischemia in the VA-ECMO group compared to the standard therapy group. Due to the COVID-19 pandemic, the study was underpowered and unable to demonstrate a statistically significant benefit of VA-ECMO, despite a numerical reduction in both 30-day and 12-month all-cause mortality. The observed survival benefit may, in part, be attributed to the concurrent use of an IABP for LV venting alongside VA-ECMO, which has been associated with improved 30-day mortality, as observed in other studies [51].

Given the lack of 30-day mortality benefits and increased bleeding risks seen in the ECMO-CS trial, the ECLS-SHOCK trial, the EURO SHOCK trial, and the meta-analysis by Zeymer et al., implementation of VA-ECMO must be targeted and used in appropriate clinical scenarios. The data from these respective studies argue against routine early VA-ECMO in CS, particularly AMI-CS, and suggest reserving VA-ECMO for refractory shock, most often in SCAI Stages D–E. Accordingly, algorithms should shift from routine early VA-ECMO toward a staged approach, using VA-ECMO as rescue therapy only after assessing response to initial stabilization and unloading, based on the clinical picture and relevant laboratory findings [48,49,50,51].

### 3.5. Protek-Duo

**Figure 5:**
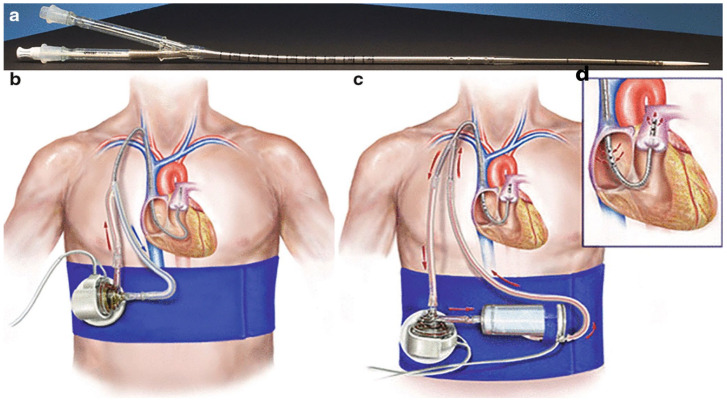
Percutaneous right ventricular assist device with (**a**) Protek Duo cannula, (**b**) without oxygenator, and (**c**) with an oxygenator, (**d**) the inflow cannulae bypassing the RV inserted directly into the PA. Arrows illustrate venous drainage (blue arrow) from the right atrium and oxygenated return (red arrow) to the pulmonary artery via the ProtekDuo cannula. Open-access image obtained from Condello et al. Percutaneous Right Ventricular Assist Device, Rapid Employment in Right Ventricular Failure During Septic Shock [101].

Protek-Duo is a dual-lumen cannula that is inserted percutaneously in the right internal jugular (RIJ) vein under fluoroscopy or ultrasound guidance. The device can support 4–5 L/min of blood flow through a centrifugal pump. As noted in Figure 5, the inflow lumen is placed in the RA, and the outflow lumen is placed in the PA, bypassing the RV. Similar to Impella RP and RP Flex, Protek-Duo is primarily for RV failure (RVF) and can be utilized with TandemHeart, Centrimag, or ECMO [56]. Its insertion into the RIJ vein, rather than the femoral vein, facilitates early ambulation in patients, allowing for earlier physical rehabilitation. ProtekDuo, similar to Impella RP and RP Flex, provides hemodynamic support in SCAI Stage C RV or BiV CS across both AMI-CS and HF-CS phenotypes [20,30,56]. Protek-Duo implementation requires expertise in imaging guidance (fluoroscopy or transesophageal echocardiography). Unlike LVADs, experience with RVADs remains limited, and the lack of standardized training highlights a need for education to safely utilize this technology [66].

From July 2016 to November 2019, Kremer et al. [99] prospectively followed 10 patients with RV-MI leading to AMI-CS, with Protek-Duo being the MCS device of choice. Significant improvements in hemodynamic parameters, such as a decrease in central venous pressure and an increase in central venous saturation, were found. Despite these findings, the study had a high 30-day mortality rate of 40%. However, the small sample size and observational design substantially limited the generalizability and strength of the conclusions. Thus, larger randomized clinical trials are needed to definitively establish the hemodynamic benefits of Protek-Duo support [99]. The Protek-Duo can also effectively support the RV following LVAD insertion, as RV failure is an often-feared complication of LVAD placement. It is a useful MCS device in managing RVF secondary to massive PE, which has a mortality rate as high as 25% [56]. In addition to providing right-sided hemodynamic support in RVF secondary to massive PE, the right IJV insertion of the device allows IVC filters to be placed more readily, preventing further thromboembolic events [56].

In RV CS secondary to acute respiratory distress syndrome (ARDS) and other pulmonologic etiologies, Protek-Duo can be utilized with Venous–Venous ECMO, a configuration known as Venous–Pulmonary ECMO (V-P ECMO). In a retrospective cohort study of 39 patients who had COVID-19 ARDS, 18 patients received V-P ECMO and 21 patients received mechanical ventilation. The V-P ECMO group, compared to the mechanical ventilation group, had significantly lower 30-day mortality and in-hospital mortality, as well as lower rates of AKI. However, the small sample size of the study limits its power and the generalizability of its promising results [100].

Moreover, Protek-Duo does not come without associated risks. Common complications include vascular injury, hemolysis, iatrogenic tricuspid regurgitation, pulmonary valve dysfunction, superior vena cava syndrome, cardiac wall perforation, pericardial effusion with tamponade, infection, embolism, thrombosis, and cannula migration, which may lead to ineffective unloading of the RV [59]. Protek-Duo support necessitates systemic anticoagulation, most commonly with unfractionated heparin. Standard monitoring parameters include an ACT goal of 160–200 s or an aPTT target of 55–85 s [37].

### 3.6. Overview of Promising Technologies in Development or Early Clinical Use (e.g., PulseCath iVAC2L, NuPulse, and Others)

PulseCath iVAC2L is a new emerging MCS device that serves as a pulsatile LVAD. It utilizes the IABP console and is inserted into the femoral artery percutaneously as the catheter is placed directly into the LV. During ventricular systole, the pump generates negative pressure, aspirating blood from the LV and storing it in the pump’s membrane chamber. Subsequently, in diastole, the pump creates positive pressure, ejecting the stored blood out of the membrane, which accumulates against a closed aortic valve, increasing coronary perfusion. The remaining blood volume is part of the next systolic output. In terms of the electrocardiogram (ECG), aspirative deflation is synchronized to the start of the QRS complex. On the arterial pressure waveform, aspirative deflation aligns with the upstroke of the waveform. In terms of diastole, ejective inflation of the balloon occurs during the T wave and coincides with the dicrotic notch of the arterial pressure waveform [102].

The iVAC2L registry retrospectively analyzed 293 patients who required iVAC2L during HRPCI. The primary endpoint was all-cause hospital mortality, while composite secondary endpoints included major adverse cardiovascular events (MACEs), significant vascular complications, major bleeding events, AKI, and episodes of severe hypotension (defined as MAP < 60 mmHg for over 10 min) during circulatory support [98]. MACEs include cerebrovascular events, as well as AMI requiring repeat revascularization. The average age of the study population was 71, and 85% of the participants were male [103]. The results of the study were promising: all-cause mortality was 1.0%, MACEs occurred in 3.1%, severe hypotension in 8.9%, major bleeding in 1.0%, and major vascular complications in 2.1%. Additional adverse event rates included AMI (0.7%), cerebrovascular events (1.4%), and cardiac arrest (1.7%). Overall, the iVAC2L has provided hemodynamic support with a good safety profile and lower risk of adverse events [103].

Nupulse iVAS (intravascular ventricular assist system) is another innovative pulsatile minimally invasive MCS device that can be used in CS or HRPCI. Generally, it combines the functions of an IABP and LVAD. The device is inserted through the subclavian artery and placed in the descending aorta. Like IABPs, the iVAS inflates during diastole to increase diastolic pressure and increase coronary perfusion [104]. Its subclavian insertion facilitates ambulation, and the device can be utilized for multiple weeks. In a 2017 prospective, single-arm, non-randomized trial by Jeevanandan et al., Nupulse iVAS was implemented in 13 patients with advanced heart failure. The primary endpoints were survival until HT or stroke-free survival at 30 days. All patients were ambulatory after 24 h, with no intraoperative complications. Patients remained in the ICU for an average of 6 ± 6 days, and all patients underwent HT approximately 32 ± 21 days post-implantation. No deaths or thromboembolic events were reported, although one patient required escalation of MCS. Despite these promising findings, further multicenter studies must be completed to gain more insights into the efficacy of using Nupulse iVAS in advanced stages of HF, CS, and HRPCI [105].

In early feasibility use, Magenta pVAD (Magenta Elevate) has emerged as a promising next-generation percutaneous LV-assist system. In the first in-human study of 14 patients undergoing high-risk PCI (including prolonged left main occlusions), the device delivered full CO support via a low-profile 9–10 French catheter, providing high output with a smaller vascular footprint compared with traditional Impella systems. There were no major device-related adverse events and no significant 30-day mortality, and during prolonged coronary occlusion, the pump maintained an MAP and aorto-left ventricular pressure differential within normal limits while unloading the LV. These features support the potential of Magenta pVAD as an emerging tool for temporary MCS, particularly in scenarios requiring high flow but with constrained vascular access [106].

Similarly, the Supira pVAD system is a novel low-profile, high-velocity continuous-flow system that is intended for use in HRPCI and CS. Placed percutaneously by utilizing a 10 Fr catheter, opposed to a 14 Fr catheter, commonly used during insertion of comparable Impella devices, the Supira pVAD system is capable of supplying up to 5.5 L/min of blood flow. Its low profile reduces the risk of limb ischemia and other vascular complications that are commonly associated with other MCS devices. The Supira pVAD system utilizes sensors to measure ventricular and aortic pressures in real time. In the first in-human (FIH) prospective, single-arm, single-center study, six patients underwent HRPCI with Supira pVAD support. In the preliminary study, the device demonstrated a 100% procedural success rate, defined by safe implantation, effective support during PCI, and successful withdrawal without major device-related adverse events (MAEs). MAEs included vascular complications, valvular injuries, or embolization. Furthermore, there were no early signs of clinically significant hemolysis. The patients had a mean age of 58 ± 9 years, were predominantly male (83%), and had a mean BMI of 30 ± 2. The common comorbidities included prior myocardial infarction (66%), diabetes (50%), tobacco use (50%), and pulmonary hypertension (50%). LVEF ranged from 30 to 60%, and 66% of the patients had multivessel disease. Device support durations ranged from 42 to 96 min. While the Supira pVAD is still undergoing trials to determine long-term efficacy and broader feasibility, early results indicate promise for clinical application [107].

## 4. Patient and Device Selection

Management of CS can be conceptualized through multiple frameworks, each shaped by factors such as shock severity, underlying etiology, anatomical considerations, and patient comorbidities. Given the high mortality rates associated with CS (20% in HF-CS and 40% in AMI-CS), stratifying patients based on these variables enables timely and tailored treatment [19]. Individualized device selection, guided by SCAI shock stage, etiology, anatomical factors, comorbidities, and institutional resources, can optimize management and improve outcomes. Device selection is discussed in the following sections.

### 4.1. Etiology of Shock

As discussed, etiologic considerations influence the modality of MCS treatment to provide the best outcome for patients suffering from CS. In AMI-CS, PCI is the first-line treatment for AMI-CS, as supported by the results of the CULPRIT-SHOCK Trial, with adjunct MCS devices. By 2020, up to 70% of patients with AMI-CS received intra-aortic balloon pump (IABP) therapy, making it the most commonly used MCS modality [31,74]. Although IABPs remain the most widely deployed form of MCS in CS, current ACC/AHA guidelines assign it a Class IIb or Class III recommendation in AMI-CS, based on shock severity, due to the absence of consistent 30-day mortality benefits, particularly in advanced stages (SCAI C–E) [29]. However, in a retrospective multicenter study of 5343 patients with AMI-CS, IABP placement significantly reduced 30-day mortality in early-stage shock (SCAI Stages A–B) to 1.2% compared to 5.8% without an IABP. Conversely, no mortality benefit was observed in advanced-stage shock (Stages C–E), where IABP use was associated with a non-significant increase in mortality (*p* = 0.172) [29]. Collectively, these findings suggest that while IABPs may confer benefits in early-stage AMI-CS, their utility diminishes in more advanced stages of shock.

The cVAD registry and DanGer Shock trial both showed a reduction in 30-day mortality in left-sided Impella in AMI-CS 30 min prior to PCI [76]. Currently, the STEMI-DTU trial is an ongoing clinical trial that is further exploring the benefits of LV unloading via Impella prior to PCI [72]. In AMI-CS, particularly in the setting of LV shock, Impella has the strongest survival benefit compared to other MCS devices. However, more studies need to be completed to further compare efficacy among MCS devices [9,108]. Impella RP and Protek-Duo can be considered in RV-predominant CS (SCAI Stages C–D), as shown by multiple studies, including the RECOVER Right Trial and Kremer et al., respectively [19]. VA-ECMO is commonly used in severe stages of AMI-CS (SCAI Stages D-E) despite increased risk of bleeding, with no significant 30-day mortality benefit, per the ECLS-SHOCK Trial [50,108]. Involving a multidisciplinary team (interventional cardiologists, heart failure specialists, cardiothoracic surgeons, intensivists) alongside hemodynamic monitoring (e.g., PAC and Right Heart Catheterization [RHC]) can tailor treatment plans accordingly for AMI-CS [109].

In HF-CS, MCS is used to stabilize patients who remain hemodynamically unstable despite initial optimization with medical therapy, including inotropes and vasopressors. MCS serves as a bridge to recovery and a bridge to decisions, including HT or durable LVAD placement. Patients who present with HF-CS typically have longer hospital stays, higher rates of MCS implementation, and HT compared to other etiologies of CS [11]. Given the dearth of clinical data in managing HF-CS specifically, the principles guiding escalation and de-escalation of pharmacotherapy and MCS in AMI-CS have been applied to HF-CS as well [110]. IABPs can be considered for early stages of CS (SCAI Stages A–B) whereas Impella 5.5 can be used in more advanced stages of LV HF-CS (SCAI Stages C–D) [111]. RVADs, such as Protek-Duo and Impella RP, can be utilized for right-sided support in SCAI Stages C–D [73,99]. If full cardiopulmonary support is required, VA-ECMO can be implemented, typically in refractory SCAI Stage D or E shock [72].

Myocarditis is defined as inflammation of the myocardium, typically caused by viral infections (e.g., EBV, Parvovirus B19, Coxsackie B3, Cytomegalovirus, COVID-19, and HIV), but it can also result from bacterial infections (e.g., Chlamydia Pneumonia, Mycobacterium Tuberculosis, Neisseria meningitidis), autoimmune diseases (e.g., Sjogren’s syndrome, systemic lupus erythematosus (SLE), rheumatoid arthritis, scleroderma), or drug toxicity (e.g., anthracyclines, checkpoint inhibitors, cephalosporin, and clozapine) [112,113,114,115,116,117,118]. Myocarditis is typically diagnosed clinically but also by endomyocardial biopsy and cardiac MRI [119]. Fulminant myocarditis, a sequela of myocarditis, has a mortality rate as high as 50–70% without MCS support due to imminent circulatory collapse [120]. While the precise pathophysiology of fulminant myocarditis leading to circulatory compromise is unknown, cytokine storm, abnormal myocardial contraction, and arrhythmia are proposed to be the leading causes [119]. Initial management of myocarditis-induced CS includes trial of inotropes and vasopressors and treating the underlying cause of myocarditis by utilizing corticosteroid therapy or immunoglobulin therapy. However, given the rapid deterioration in fulminant myocarditis, MCS initiation is often required to stabilize patients [119].

MCS has played a critical role in managing fulminant myocarditis by serving as a bridge to recovery or a bridge to decision; if it cannot be weaned within 2 to 3 weeks, long-term options, such as LVAD placement or HT, are typically considered at advanced stages [121,122]. If isolated LV dysfunction is suspected, a left-sided Impella or an IABP may be initiated first. In cases where RV dysfunction is suspected, RVADs such as the Impella RP or Protek-Duo can be considered. If additional hemodynamic support is needed, VA-ECMO can be added to an existing left-sided MCS device. As noted previously, VA-ECMO is often used in conjunction with an IABP or a left-sided Impella to reduce LV afterload [121]. In a retrospective study from 2007 to 2013, Mody et al. [120] studied 11 patients with fulminant myocarditis, of whom 8 survived following MCS initiation. However, CHANGE PUMP 2 studied 216 patients with CS secondary to fulminant myocarditis receiving MCS, which indicated that VA-ECMO may have a worse prognosis for treating fulminant myocarditis than an IABP or Impella alone. The multicenter retrospective study conducted in Japan (2000–2020) compared outcomes in 216 patients with fulminant myocarditis: 155 received VA-ECMO (with or without an IABP/Impella), while 61 received an IABP or Impella alone. The composite endpoint (death, LVAD implantation, or heart transplantation) occurred more frequently in the VA-ECMO group [123]. Patients in the VA-ECMO group also had significantly higher markers of organ injury (i.e., higher creatine, aspartate transaminase, alanine aminotransferase, LDH, and creatine kinase–myocardial band) [123]. Consequently, VA-ECMO was associated with a worse prognosis in the acute phase compared to an IABP or Impella alone [123].

### 4.2. Development of Combination Therapies

Notably, hybrid strategies combining different MCS devices have become a growing area of interest in managing CS. A relatively novel strategy, the “BiPella” approach, involves the simultaneous use of both LV and RV Impella pumps to provide hemodynamic support in cases of BiV-CS. As BiV involvement is seen in up to 50% of CS cases, “BiPella” could prove to be a useful option to treat BiV CS [7,68]. A retrospective study of 20 patients with BiV CS were treated with “BiPella”. While there were statistically significant increases in CO and decreases in LV and RV filling pressures, the mortality rate was as high as 50% [7]. Given the novelty of this strategy, well-designed randomized controlled trials with larger sample sizes are required to establish its safety and efficacy.

Another approach is “EcPella”, which combines VA-ECMO with LV Impella pumps simultaneously. In the peripheral configuration, the retrograde arterial flow generated by VA-ECMO increases afterload, which can elevate LVEDP and PCWP. This, in turn, may precipitate pulmonary edema due to rising left-sided filling pressures and increased myocardial oxygen demand. Impella placement during VA-ECMO has been shown to improve LV offloading, reducing afterload and myocardial oxygen requirements while providing systemic circulatory support from VA-ECMO. In a retrospective study by Pappalardo et al. [70], 157 patients were treated with VA-ECMO for CS, of which 34 received “EcPella”. The “EcPella” group had a significantly lower 30-day mortality rate compared to the VA-ECMO alone group, with higher rates of successful bridging to recovery or further therapy, with no significant difference in bleeding risks between the two groups [70,71]. The results of these studies highlight the potential hemodynamic benefits of LV venting to mitigate VA-ECMO-induced afterload as a potential strategy in managing CS. However, the retrospective nature and limited sample sizes of these studies underscore the need for large, well-designed randomized controlled trials to more fully assess the magnitude of the hemodynamic benefit of “EcPella”.

Similar to “EcPella”, IABPs can also be utilized as a form of LV venting when combined with VA-ECMO. A multicenter retrospective study included 5492 patients on VA-ECMO from the Chinese Extracorporeal Life Support (CSECLS) registry from January 2017 to July 2023. The primary outcome was 30-day in-hospital mortality. The secondary outcomes included 30-day mortality, survival on VA-ECMO, and associated complications. Overall, IABP insertion after VA-ECMO initiation reduced in-hospital mortality and resulted in a higher survival rate on VA-ECMO. Conversely, the IABP after VA-ECMO group had more device-associated complications, such as mechanical complications, increased risk of bleeding, renal complications, and pulmonary complications. The study showed that utilizing an IABP for LV unloading with VA-ECMO reduced its deleterious effects on afterload, but it also increased risks of device-associated complications [124].

In a retrospective observational study, 3815 patients with AMI-CS were recruited from the JROAD (Japanese Registry of All Cardiac and Vascular Diseases)—DPC from April 2012 to March 2018. A total of 2964 (77.7%) patients were managed with combined VA-ECMO and IABP, and 851 (22.3%) were managed with VA-ECMO alone. The patients treated with combined VA-ECMO and IABP had significantly lower in-hospital, 7-day, and 30-day mortality compared to those receiving VA-ECMO alone. The study further underscores the possible mortality benefits of using an IABP alongside VA-ECMO for LV venting, particularly in managing AMI-CS [125].

A meta-analysis by Gandhi et al. compared “EcPella” to VA-ECMO with an IABP and found no statistically significant difference in 30-day inpatient mortality between both groups. However, the study found more complications in the “EcPella” group, such as major bleeding and hemolysis. Thus, the study showed that both “EcPella” and VA-ECMO with an IABP had similar outcomes in managing CS, but “EcPella” was associated with increased risk of complications [126].

### 4.3. Anatomical Considerations and Patient Comorbidities

Anatomical variations, such as common femoral artery diameter, presence of atherosclerosis, and mobility status, affect the selection of MCS devices [127]. Although femoral insertion of an IABP is the most common method, the intra-axial approach has proven to be beneficial in certain clinical scenarios. Typically, the intrafemoral approach is more common given that the common femoral arterial diameter is generally wider than the axillary artery. However, complex intrafemoral anatomy or extensive atherosclerotic disease, such as Leriche syndrome, in the femoral arterial system can hinder IABP placement. In these scenarios, the intra-axial approach is preferred since axillary arteries are less likely to suffer from atherosclerotic disease compared to femoral arteries [127]. Additionally, the intra-axial approach facilitates early ambulation, promoting faster physical rehabilitation. However, because axillary arteries are smaller in diameter, they require prior fluoroscopy to guide precise insertion. Insertion requires placing the guidewire into the thoracoacromial artery and lateral thoracic artery underneath the pectoralis minor. Improper placement risks causing pneumothorax or injuring the brachial plexus [128].

Protek-Duo and Impella RP Flex allow for ambulation given the IJV approach during RV or BiV failure [56]. Similarly, given the insertion into the subclavian artery, NuPulse iVAS allows for easy ambulation and forgoing the femoral arterial approach if there is the presence of extensive atherosclerosis or PAD [129]. Device placement that permits ambulation enables patients to engage more readily in physical therapy and in-hospital exercises and facilitates faster rehabilitation [130]. The transcaval approach, accessing the aorta through the IVC, can be utilized to deploy Impella 5.0 or Impella LD due to iliofemoral atherosclerotic disease or small femoral artery diameter. This approach is typically used in transcatheter aortic valve replacement (TAVR) or transcatheter endovascular aortic repair (TEVAR). Moreover, the transcaval approach reduces the risk of lower limb ischemia compared to the intrafemoral approach. The safety profile is comparable to the intra-axillary or subclavian approach when performed by experienced proceduralists. The technique involves using intracatheter electrocautery to create an aortocaval fistula using a high-frequency alternating current to vaporize the surrounding tissue. Since retroperitoneal pressure is greater relative to IVC pressure, any aortic bleeding will tamponade into the IVC primarily. Once the MCS device is deployed, the aortocaval fistula is closed by heparin reversal with protamine and placement of a nitinol occluder. The technique generally requires careful preoperative planning with CT Angiography for proper placement. However, in emergent cases, catheters in the aorta and cava can visualize insertion sites while utilizing fluoroscopy to help identify anatomical landmarks. Overall, the transcaval technique offers an alternative method for deploying large-bore MCS devices that would otherwise require surgical cutdown, an option that can be considered in patients who are deemed poor surgical candidates [131,132].

TandemHeart can be used selectively in patients who have LV thrombus, mitral stenosis, or aortic stenosis, given intraseptal LA placement, since left-sided Impella devices are contraindicated in these cases [43,44,45]. Age is an often-overlooked comorbidity during MCS placement. Elderly patients, particularly those older than 75, are more susceptible to complications from MCS placement, such as bleeding, ICU-acquired myopathy, deterioration of existing organ function, infections, and decline in cognitive function during nonpulsatile mechanical support [133]. Thus, careful selection of MCS devices for hemodynamic support is essential in this patient population, along with prudent monitoring following placement.

### 4.4. Institutional Resources and Interdisciplinary Shock Team

The selection of MCS devices is dependent on the severity of shock, availability of devices at the institution, vascular access, and overall prognosis [19]. Institutional facilities are categorized into Levels 1–3 centers for managing CS. Level 1 centers offer full support for CS, including advanced MCS devices, durable LVAD placement, and heart transplantation. Level 2 centers are typically PCI-capable but lack advanced MCS. Level 3 centers, which are often located in rural communities, only provide initial stabilization and subsequent transfer to higher-level facilities [134,135]. The “spoke–hub model” is a model in which spokes (Level 2 or 3 centers) identify and stabilize patients in CS in order to transfer them to hubs (Level 1 centers) that have PCI capabilities, advanced MCS devices, and designated shock teams to further manage care [7]. Most patients initially present to Level 3 centers, where they are typically evaluated and stabilized, before being transferred to higher-level centers (Level 1 or 2) for definitive management [136,137].

Institutional resources and team expertise play vital roles in managing CS. Per the AHA/ACC/HFSA guidelines, implementation of an interdisciplinary shock team has a Class IIa recommendation with Level B evidence [7]. Coordinating care for CS with an interdisciplinary designated shock team allows for optimizing care delivery, expediting therapeutic interventions, and integrating specialized heart failure services to prevent rapid deterioration [138]. The interdisciplinary shock team consists of interventional cardiologists, heart failure specialists, intensivists, and cardiothoracic surgeons [139]. The results from the Critical Care Cardiology Network, a multicenter study during 2017–2019 with 24 CICUs, 10 of which used multidisciplinary shock teams, found more favorable patient outcomes in CICUs that utilized shock teams. The study found that centers with shock teams used PACs more frequently and utilized more advanced types of MCS rather than IABPs, despite implementing fewer overall MCS devices. The presence of a shock team was independently associated with lower CICU mortality [108]. Overall, the study demonstrated that shock teams implemented PACs more frequently, correlating with findings from previous studies and showing the mortality benefits of invasive hemodynamic measurements in patients with CS. Additionally, it showed that centers with designated shock teams often implemented advanced MCS devices and had reduced CS mortality compared to centers without a shock team [140]. Similar findings were reported in the Utah Cardiac Recovery Shock Team study, which prospectively enrolled 123 patients with refractory CS managed by a dedicated shock team between April 2015 and August 2018 and retrospectively compared outcomes in 121 patients treated without a designated shock team. Patients managed by a dedicated shock team had improved outcomes overall [108].

### 4.5. Decision-Making Algorithms for Optimal Device Choice

**Figure 6:**
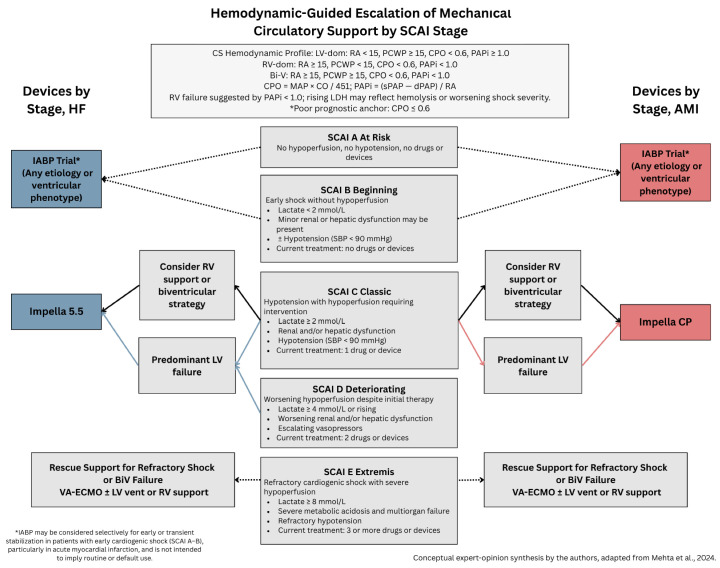
Conceptual hemodynamic-guided algorithm for escalation of mechanical circulatory support in CS. This figure represents an expert-opinion conceptual synthesis by the authors and is adapted from contemporary shock team-based protocols and related registry data [20]. This synthesis is not intended to serve as a formal clinical guideline. Escalation pathways may vary based on institutional expertise, available resources, and patient-specific hemodynamic profiles. In this schematic, device examples are shown to reflect common clinical practice patterns, with HF-CS emphasizing higher-flow axial support (e.g., Impella 5.5) and AMI-CS emphasizing percutaneous axial support (e.g., Impella CP). Arrows (dotted and solid) are used as visual guides to orient the reader from each SCAI stage to representative mechanical circulatory support (MCS) strategies shown laterally in the figure. Dotted and color-coded arrows are intended to facilitate visual flow and emphasize typical MCS utilization patterns within a given SCAI stage based on hemodynamic phenotype and clinical context. Arrow directionality is not intended to represent mandatory escalation, inter-stage transitions, or prescriptive treatment pathways. *Intra-aortic balloon pump (IABP) placement in early cardiogenic shock (SCAI A–B) reflects historical and selective use within shock team–based protocols for initial or transient stabilization, particularly in acute myocardial infarction, and is not intended to imply routine or guideline-directed use. This schematic is intentionally simplified to highlight dominant physiologic clinical flow and patterns of mechanical circulatory support utilization. Other temporary MCS devices, including TandemHeart, Protek Duo, and alternative ventricular assist configurations, remain appropriate in selected clinical scenarios based on institutional expertise and patient-specific anatomy.

Optimal MCS device choice is guided by the severity of shock, available institutional resources, operator expertise, and patient comorbidities [138]. Algorithms, such as the one created by the Inova task force, as described earlier, are based on SCAI shock stage and ventricular pathology, allowing clinicians to make streamlined decisions readily [20]. Figure 6 illustrates a stage-based management framework that integrates shock etiology, ventricular phenotype, and SCAI stage classification to guide individualized MCS deployment, device selection (LV, RV, BiV), and bridge strategy (bridge to recovery [BTR] vs. bridge to transplantation [BTT]) within a multidisciplinary shock team.

### 4.6. Weaning Strategies for MCS Devices

Weaning from MCS is essential for recovery in CS, but no randomized trials currently guide practice. Strategies rely on institutional protocols, clinical judgment, and observational data. Per AHA guidance, readiness for weaning includes evidence of myocardial recovery, minimal or discontinued inotropes/vasopressors, and improved end-organ perfusion (lactate, creatinine, aminotransferases) [138]. Maximum vasopressor/inotrope doses in DanGer Shock prior to weaning include dobutamine 10 µg/kg/min, dopamine 10 µg/kg/min, milrinone 0.4 µg/kg/min, and norepinephrine 0.15 µg/kg/min [9]. Hemodynamic markers, CVP, CI, CPO, PCWP, and PAPi, should be monitored during down-titration [138].

Device-specific weaning includes the following:

- IABP: transition from 1:1 to 1:2, then 1:3 support prior to decannulation.
- Impella: reduce P-levels by 1–2 every 2–4 h (max P9), with decannulation at P2.
- TandemHeart, Protek-Duo, VA-ECMO: decrease flow by 0.5 L/min every 2–4 h until ~2 L/min, then decannulate [138].

In general, AMI-CS tolerates more rapid de-escalation, whereas HF-CS requires a slower weaning approach [138].

## 5. Ongoing Research and Future Directions

### 5.1. Overview of Ongoing Trials and Registries

Given the persistently high mortality associated with CS, MCS devices have become a cornerstone of management. Consequently, the evidence-based evaluation of their efficacy continues to evolve and expand. A multitude of clinical trials and multicenter studies are currently being conducted or are in ongoing completion to further elucidate the benefits of MCS deployment. As emerging data refine current understanding, a more targeted and individualized approach, particularly emphasizing the potential benefits of early initiation, is anticipated to guide future practice.

### 5.2. Prospective and Ongoing Trials

The PROTECT IV Trial is an ongoing randomized controlled trial investigating the outcomes of Impella CP or 2.5 in patients with reduced LVEF during HRPCI. The main objective of the study is to evaluate whether Impella-supported HRPCI improves long-term outcomes—specifically, three-year rates of death, stroke, myocardial infarction, repeat revascularization, LVAD implantation, heart transplantation, and cardiovascular hospitalizations—compared with HRPCI performed without Impella support [141].

The Assessment of ECMO in Acute Myocardial Infarction Cardiogenic Shock (ANCHOR) is an ongoing randomized observational study conducted in Paris, France, with both a retrospective and prospective cohort comparing the outcomes of VA-ECMO with an IABP to medical treatment alone in treating AMI-CS. As a rescue option, patients in the medical treatment arm who experience clinical deterioration, such as persistent CS, unstable arrhythmias, or cardiac arrest, are crossed over to the VA-ECMO with an IABP arm. The primary outcome of the study is treatment failure at 30 days, defined as death in the VA-ECMO group and the rescue VA-ECMO group [142]. Secondary outcomes at both 30 days and 1 year include MACEs, stroke, recurrent MI, need for repeat PCI or CABG, renal replacement therapy, cardiac transplantation, and LVAD placement [142]. The study aims to recruit 400 participants.

The STEMI-DTU trial is an ongoing prospective, randomized multicenter trial evaluating whether initiating LV unloading with Impella CP for 30 min before PCI improves outcomes compared to performing PCI without prior LV unloading in patients with anterior STEMI [72]. The primary endpoint is determined by LV infarct size via cardiac MRI (CMR) 3–5 days after PCI. The secondary endpoints include infarct size as a percent of LV mass at 3–5 days after PCI, bleeding complications at 30 days after PCI, and LVAD or HT at 12 months. Follow-up is planned for 5 years [72]. Thus far, the study has recruited 527 participants.

The SWISS Circulatory Support Registry (CARDSUP) is an ongoing registry that is prospectively collecting clinical procedural data on VA-ECMO and Impella implementation across various sites in Switzerland. The devices chosen for study are Impella CP, 2.5, 5, or RP and VA-ECMO. All participants are prospectively registered on specific devices; however, implementation is performed at the discretion of the physician. The primary outcome measured is all-cause mortality at 30 days. The secondary outcome measures at 6 months include MACE, vascular complications, bleeding, duration of MCS implementation, NYHA Class, and mortality at 6 months [143]. The registry is aiming to recruit 1500 participants.

These prospective studies increasingly demonstrate that early and timely initiation of MCS confers significant clinical benefit, including reductions in mortality. Collectively, the findings of these studies hope to further expand the knowledge of MCS management and inform future guidelines and standards of care in CS. Further and ongoing trials are critically important due to persistent gaps in evidence for the management of CS, in which there are limited data and interventions for a syndrome that still retains a high mortality rate.

### 5.3. Innovations in Patient Monitoring and Predictive Analytics (e.g., Artificial Intelligence (AI)-Based Decision Support)

Beyond clinical trials, innovations in patient monitoring and predictive analytics continue to advance. Artificial Intelligence (AI), whilst in its early stages of development, is an emerging aspect in managing CS. AI can guide MCS deployment by using clinical parameters such as hemodynamic measurements, laboratory results, and patient demographics to personalize MCS device selection and timing. The AHA recommends timely CS intervention to prevent further deterioration, with AI diagnostic tools potentially supporting clinicians in providing timely MCS escalation more widely in the future [138,144].

AI-based decision support with machine learning has been promising in predicting the onset of CS. A systematic review by Aleman et al. examined 943 studies with a cohort of 698 patients and demonstrated that the area under the (AUC) receiver operating characteristic (ROC) curve had a value of 0.82 (α = 0.05), indicating strong sensitivity and specificity for detecting CS. However, 159 deaths (22.8%) were identified following early CS detection [145].

Another notable study, completed by Obmann et al. [146], retrospectively studied patients admitted to the critical care units in the Beth Israel Deaconess Medical Center in Boston from 2001 to 2012 via the Medical Information Mart for Intensive Care (MIMIC) III database. A total of 5970 patients were analyzed and classified as having sepsis, septic shock, or CS [146].

Bayesian Network Classifiers (BNCs) were used to differentiate between all three diagnoses with vasopressor dependency, lactate levels, SOFA scores, and infection status. However, sensitivities and specificities in detecting CS were lower than those in detecting sepsis or septic shock. Additionally, concomitant conditions, such as sepsis and AMI, made it more difficult to differentiate between the conditions. While the study shows promise for AI-based decision-making utilizing BNCs, it underscores the need for further advancements in refining AI guidance in clinical decision-making regarding CS [146]. Ultimately, patient care relies on the clinician’s judgement, integrating available biomarkers and the patient’s clinical presentation to tailor individualized management strategies and optimize outcomes in CS.

The Impella SmartAssist system leverages live hemodynamic data to guide escalation, weaning, or de-escalation of support in real time. AI modules for these systems are continually evolving, incorporating hemodynamic parameters, laboratory results, and echocardiographic findings to optimize management. One emerging concept is the “virtual twin,” a simulated patient clone generated from a defined monitoring period to model and assess potential treatment strategies, enabling clinicians to evaluate outcomes without direct patient intervention. However, such models cannot fully replicate dynamic physiological responses to vasopressors or MCS devices [147]. Additionally, the Impella Connect network aggregates multicenter data to train machine learning algorithms that may, in the future, enhance prediction and prevention of complications, such as hemolysis, limb ischemia, and AKI, as well as refine decisions regarding timing and duration of MCS therapy.

## 6. Interpretation of Evidence, Bias, and Resource Utilization in Mechanical Circulatory Support

### 6.1. Comparative Synthesis of MCS Devices

Deployment of MCS devices is informed by clinical necessity, particularly the required flow rate, timing of deployment, and the technical expertise needed for device insertion. The IABP remains the most accessible and cost-efficient form of MCS due to its bedside placement and favorable safety profile [29]. Although the IABP-SHOCK II trial did not demonstrate a 30-day mortality benefit compared with PCI alone, IABPs have been shown to improve hemodynamic parameters such as MAP, CO, and CPO [31]. With modest hemodynamic support of approximately 0.5 L/min, retrospective multicenter studies support its use primarily in earlier stages of CS (SCAI Stages A–B) [29]. Additional hemodynamic effects include afterload reduction and enhanced diastolic coronary perfusion, contributing to improved MAP.

Impella micro-axial pumps provide substantially higher flows—ranging from 2.5 L/min to nearly 6 L/min depending on the device model—via active ventricular unloading [35,36,37]. These features make Impella preferable to IABPs in SCAI Stage C–D AMI-CS, where rapid augmentation of forward flow is required. However, despite superior hemodynamic support, Impella use has been consistently associated with higher bleeding risk and, in select observational analyses, increased mortality relative to IABPs [74]. Thus, although LV Impella offers greater support in SCAI Stage C–D left-sided CS, its use warrants caution given its complication profile and the absence of definitive survival benefit to date [74]. Additional randomized trials in NSTEMI-CS and HF-CS populations are required to clarify its therapeutic benefit [18].

TandemHeart delivers 3.5–5 L/min of centrifugal flow via left atrial drainage and arterial reinfusion. Compared with IABPs, TandemHeart produces more robust hemodynamic improvements, including higher CI and MAP and reduced PCWP, yet randomized trials have shown neutral 30-day mortality outcomes [79]. Because of its superior circulatory support, TandemHeart is favored over IABPs in refractory SCAI C–D LV-predominant shock. Nevertheless, its adoption remains limited due to the technical demands of transseptal cannulation, higher procedural risk, and more intensive anticoagulation requirements [60,79,82,148]. A potential niche indication is the presence of LV thrombus, which precludes LV Impella placement and may favor transseptal LA drainage [41,148]. Larger randomized trials are needed to define the relative efficacy of TandemHeart, particularly in patients with LV thrombus.

VA-ECMO provides the highest degree of circulatory support, typically 4–7 L/min and up to approximately 7–8 L/min depending on cannula size, enabling near-complete cardiopulmonary bypass [48,49,50,51]. These high flows also allow support of advanced BiV failure [87]. However, before initiating VA-ECMO, lower-flow MCS should be considered, as both ECLS-SHOCK and ECMO-CS demonstrated no mortality advantage with routine early ECMO in AMI-CS. Current evidence supports reserving VA-ECMO for refractory SCAI Stage D–E shock [48,49,50,51]. Combination strategies, such as LAVA-ECMO and “EcPella”, are being explored to mitigate the afterload elevation and pulmonary congestion intrinsic to ECMO, though available data remain observational [70,71,149]. Randomized trials are needed to determine the clinical value of these hybrid unloading strategies.

For isolated RV-predominant CS (SCAI Stages C–D), Protek-Duo and Impella RP provide comparable maximum flows and represent commonly used percutaneous RV support modalities [35,36,56]. Registry comparisons suggest increased use of Protek-Duo in HF-CS with isolated RV failure, although higher bleeding rates and longer hospital stays have been observed [150]. The RIJ insertion of Protek-Duo facilitates early ambulation, a capability that is now also available with Impella RP Flex. Additional randomized trials are required to compare outcomes across isolated RV-shock phenotypes.

These comparative physiologic and operational differences across platforms also help explain their divergent RCT outcomes. IABP-SHOCK II likely failed not because counterpulsation lacks physiologic benefit but because the modest flow augmentation of IABPs is inadequate in SCAI Stage C–E shock, where systemic inflammation and multiorgan dysfunction dominate the clinical trajectory [31]. DanGer-Shock demonstrated benefit where prior Impella studies did not largely because it enrolled a highly selected anterior STEMI-CS population, mandated ultra-early unloading, excluded patients at the highest risk of futility, and was conducted in high-volume centers with extensive Impella experience [9]. Conversely, VA-ECMO trials, such as ECLS-SHOCK and ECMO-CS, showed neutral survival effects likely because ECMO’s hemodynamic benefits were counterbalanced by increased afterload, bleeding, limb ischemia, and low rates of adjunctive LV unloading [48,49,50,51]. Across all platforms, these findings illustrate that even dramatic hemodynamic improvement may possibly fail to translate into mortality benefit when device-related complications, delayed deployment, or established multiorgan dysfunction outweigh flow-based support.

### 6.2. Key Methodological Challenges in MCS Research

Interpretation of MCS evidence is constrained by recurring methodological biases. Confounding by indication is pervasive because patients with higher lactate, escalating vasopressors, or post-arrest physiology are preferentially selected for advanced devices, as shown in Dhruva et al.’s JAMA cohort of >28,000 AMI-CS patients and in the cVAD registry, where major baseline differences persist despite adjustment [74,76]. Survivor bias further skews outcomes, as patients must live long enough to receive MCS; in DanGer-SHOCK, only individuals surviving to meet strict enrollment thresholds were randomized, excluding the sickest early-mortality patients [9]. Immortal time bias affects timing studies (particularly early-versus-late Impella comparisons) because patients labeled “early” must survive the pre-implant interval, while early deaths accumulate in the “late” or no-device cohorts, exaggerating apparent benefit [151]. Selection bias is also substantial in DanGer-SHOCK, which enrolled a highly selected anterior STEMI-CS population from experienced European centers, limiting generalizability to broader AMI-CS and HF-CS phenotypes [9,18].

Together, these biases explain why MCS studies consistently show robust hemodynamic improvement yet inconsistent survival effects. The gap between physiologic and clinical benefit reflects not only device-related complications and timing of deployment but also structural methodological limitations that complicate the interpretation and comparison of MCS outcomes across platforms.

### 6.3. Economic Burden

The financial and economic burden of MCS devices vary significantly by device, patient, and clinical outcomes. The IABP is the most cost-effective, with an estimated per-patient cost of USD 800 [146]. In contrast, the Impella device carries substantially higher costs, ranging from USD 20,000 to USD 25,000. However, its use may reduce downstream costs by shortening ICU stays, lowering the incidence of hospital complications, and reducing the need for repeat revascularization procedures. The cost per added quality-adjusted life year (QALY) remains under USD 100,000 which is commonly accepted in the U.S. threshold for cost-effectiveness [152].

Direct per-patient expenses for temporary MCS vary by device, although the hardware itself typically represents only a portion of overall cost. For TandemHeart, the disposable cannulae and console use create a direct device expense that is generally in the tens of thousands of dollars per case, whereas the direct hardware cost of VA-ECMO is comparatively modest because the pump, oxygenator, and cannulas are less expensive than the downstream care they necessitate. Consistent with prior expert consensus statements, the major cost drivers for VA-ECMO are related to the clinical setup rather than the circuit itself, including prolonged ICU care, continuous hemodynamic and anticoagulation monitoring, specialized personnel, and management of complications [41]. Episode-of-care analyses further demonstrate that VA-ECMO results in substantially higher total costs than other percutaneous VAD support, primarily due to longer length of stay and increased resource utilization rather than device hardware price [153]. These resource-intensive requirements are shared across advanced MCS modalities.

### 6.4. Strategies to Optimize Resource Utilization

Given the substantially higher costs associated with Impella and VA-ECMO, their use should be reserved for patients in need of more intensive and aggressive intervention. Although IABPs are considerably less expensive, their limited impact on mortality raises questions about its cost-effectiveness in many clinical scenarios. In only 15.2% of eligible cases, MCS is employed, and fewer than 1% of patients receive Impella or ECMO support.

The long-term costs associated with MCS extend far beyond initial hospitalization and placement of the MCS device. These costs are associated with significant post-discharge disability, rehabilitation services, long-term care facilities, and recurrent hospital readmissions. These costs are especially increased in patients with multiple comorbidities. In order to optimize resources, clinical strategies should not solely focus on survival but also aim to minimize long-term functional decline. Studies have shown that patients who spend more time at home following hospital discharge obtain significantly lower total healthcare costs, which emphasizes the importance of early and appropriate device selection, with comprehensive post-discharge care planning [154].

## 7. Conclusions

The management of CS has undergone a significant transformation with the advent of percutaneous MCS devices. From an IABP and Impella to VA-ECMO and Protek-Duo, clinicians now have a diverse arsenal to tailor CS management based on shock severity, etiology, and ventricular involvement. The integration of SCAI staging, invasive hemodynamic monitoring, and multidisciplinary shock teams has further refined diagnosis, risk stratification, escalation strategies, and outcomes.

Emerging devices, such as the PulseCath iVAC2L, NuPulse iVAS, Magenta pVAD, and Supira pVAD, demonstrate the potential to expand MCS use with improved safety profiles and enhanced mobility. As trials like STEMI-DTU, ANCHOR, and PROTECT IV progress, they suggest a shift toward earlier, more targeted support strategies that may improve both short-term and long-term survival. Innovative combined MCS strategies, such as “EcPella” and “BiPella”, offer potential new approaches to CS management; however, further studies are required to establish their safety and efficacy. The development of risk stratification scores helps clinicians weigh the benefits of MCS deployment against its risks, tailoring individualized treatment plans.

The strength of evidence supporting each MCS device remains heterogeneous. IABPs have been evaluated in multiple randomized controlled trials, including IABP-SHOCK II, which demonstrated hemodynamic improvement without a significant 30-day mortality benefit in advanced shock (SCAI Stages C–E) [31]. However, retrospective observational data suggest a potential mortality benefit when used in earlier stages of CS (SCAI Stages A–B), irrespective of the underlying ventricular insult or CS phenotype [31]. Impella micro-axial flow pumps have been extensively studied through large registries and the pivotal DanGer-Shock trial, yet observational analyses continue to raise concerns regarding bleeding and mortality [72,73]. Current evidence supports Impella use primarily in SCAI Stage C–D CS for both LV and RV failure [9,36,37,66]. TandemHeart and Protek-Duo provide substantial hemodynamic support, but existing data remain limited to observational cohorts; available evidence suggests their use in SCAI Stage C–D CS, with TandemHeart favored for LV failure and Protek-Duo for RV failure [43,44,45,56]. VA-ECMO has undergone the most rigorous randomized evaluation, including ECMO-CS and ECLS-SHOCK, both of which reported no significant mortality benefit, particularly in AMI-CS [48,49,50,51]. Contemporary practice therefore reserves VA-ECMO for refractory, advanced-stage shock (SCAI Stages D–E), regardless of phenotype or ventricular involvement [50,109].

Despite these advances, significant knowledge gaps remain. Comparative efficacy between devices, anticoagulation strategies, optimal timing of initiation, and long-term outcomes needs to be better defined. Moving forward, collaborative research efforts, real-world data registries, AI-based decision-making, and innovation in device technology will be essential to fully realize the potential of MCS in CS. The future of CS management lies not only in expanding the current technological capabilities but in unifying them within evidence-based, patient-centered frameworks.

## Data Availability

No new data were created or analyzed in this study.

## Appendix A

**Table A1:** Hemodynamic parameters in cardiogenic shock.

Parameter	Definition/Formula	Normal Range	Clinical Relevance in CS
Mean Arterial Pressure (MAP)	(SBP + 2 × DBP)/3	70–100 mmHg	MAP < 60 mmHg indicates inadequate perfusion.
Cardiac Output (CO)	Heart Rate × Stroke Volume	4.0–8.0 L/min	Typically reduced in CS due to impaired ventricular function.
Cardiac Power Output (CPO)	(MAP × CO)/451	>0.8 watts	CPO < 0.6 watts suggests severe LV dysfunction.
Pulmonary Artery Pulsatility Index (PAPi)	(PA Systolic—PA Diastolic)/Mean RA Pressure	>1.5	PAPi < 1.0 suggests RV failure.
Pulmonary Capillary Wedge Pressure (PCWP)	Direct catheter measurement via PA catheter	6–12 mmHg	Elevated in LV failure; often >15 mmHg in CS.
Right Atrial Pressure (RAP)	Direct measurement via central venous or PA catheter	2–8 mmHg	Elevated in RV or BiV failure.
Cardiac Index (CI)	CO/Body Surface Area (BSA)	2.5–4.0 L/min/m^2^	CI < 2.2 L/min/m^2^ indicates shock state.
